# Uncovering the superior corrosion resistance of iron made via ancient Indian iron-making practice

**DOI:** 10.1038/s41598-021-81918-w

**Published:** 2021-02-19

**Authors:** Deepak Dwivedi, Jitendra P. Mata, Filomena Salvemini, Matthew R. Rowles, Thomas Becker, Kateřina Lepková

**Affiliations:** 1 Curtin Corrosion Centre, WA School of Mines: Minerals, Energy and Chemical Engineering, Faculty of Science and Engineering, Curtin University, Perth, Australia; 2 Australian Centre for Neutron Scattering, Australian Nuclear Science and Technology Organisation (ANSTO), Lucas Heights, NSW 2234 Australia; 3 John de Laeter Centre, Curtin University, Perth, Australia; 4 School of Molecular and Life Sciences (Chemistry), Curtin Institute for Functional Molecules and Interfaces, Faculty of Science and Engineering, Curtin University, Perth, Australia

**Keywords:** Metals and alloys, Corrosion, Characterization and analytical techniques

## Abstract

Ancient Indian iron artefacts have always fascinated researchers due to their excellent corrosion resistance, but the scientific explanation of this feature remains to be elucidated. We have investigated corrosion resistance of iron manufactured according to traditional metallurgical processes by the Indian tribes called ‘Agaria’. Iron samples were recovered from central India (Aamadandh, Korba district, Chhattisgarh). Iron artefacts are investigated using a range of correlative microscopic, spectroscopic, diffraction and tomographic techniques to postulate the hidden mechanisms of superlative corrosion resistance. The importance of manufacturing steps, ingredients involved in Agaria’s iron making process, and post-metal treatment using metal-working operation called hot hammering (forging) is highlighted. This study also hypothesizes the probable protective mechanisms of corrosion resistance of iron. Findings are expected to have a broad impact across multiple disciplines such as archaeology, metallurgy and materials science.

## Introduction

The Indian subcontinent has been famous among metallurgists, conservators and archaeologists for its ancient iron-making technological heritage^1^. Juleff et al. (1996) have highlighted the importance of South Asian iron and steel making technology, particularly in peninsular India during the first millennium AD^2^. Significant metallurgical achievements have been made in Indian subcontinent such as the discovery and application of *wootz* steel (also called “*bulad*” in Central Asia)^3,4^. Solid-state ore reduction was one of the important iron-smelting technologies applied in the iron making. This process generates agglomerated bloom as the end-product along with slag^2^. Iron-based archaeometallurgical artefacts are important in demonstrating the ancient technological developments that proceeded and dispersed in the ancient times^5,6^. The Indian blacksmiths were famous globally and the steel (*wootz* steel—produced from iron bloom manufactured by Indian tribes) produced by them was used in Damascus steelmaking, which was famous for its excellent mechanical properties^7^. Indian ancient metallurgical and iron making practices have attracted significant attention in the recent past but detailed investigations on the structural properties of the tribe’s made iron are yet to be performed^8^.

The ancient Indian iron-making technology has been famous for producing corrosion-resistant iron. The Delhi iron pillar, which was made around fifth century AD (~ 1600 years old) is famous for its immunity to rusting and a prominent example of the Indian iron making process^9,10^. The Delhi iron pillar is also an important example of tribal tradition (Indian blacksmiths) of iron making in India. Several theories have been postulated regarding corrosion resistance of the Delhi iron pillar. Some of those refer to the inherent nature of the construction material, such as the selection of pure iron, presence of slag particles and slag coatings, surface finishing using mechanical operation, phosphate film formation, or the Delhi’s climate^11–17^. The exact reason for the superior corrosion resistance phenomenon remains a mystery. It is not possible to examine the iron from the Delhi Iron pillar as it is an archaeologically protected monument.

We have examined iron that was manufactured by Indian blacksmiths (Agaria tribes) using an Indian traditional technique. Agaria tribes are regarded as important tribal community responsible for channelizing the traditional iron and steel making technological growth in various Indian states in the central region, such as Madhya Pradesh and Chhattisgarh, Orissa Eastern part of Uttar Pradesh and Bihar from the ancient times^18^. It is noteworthy that Agaria tribes were not allowed to reside near to the villages, hence they used to perform iron making practice in a deep forest and adopted this practice as their means of livelihood. They were famous for performing special rituals customs (i.e. worshipping their God called *Lohasur*) prior to the start of iron-making^19^. India’s status as a colony of the British Empire until 1947, affected the traditional iron-making practices that were banned by the British rulers. Therefore, the technological and historical knowledge of ancient iron-making by Agaria tribes was lost and only a few clues can be obtained from the travel documents written earlier. There are still a few old members of the same tribes who have seen iron making practices in their early childhood. These are the only remaining sources of the iron making knowledge other than the travel documents. Agaria tribes’ soundness and skills in iron making technology made them travel to Japan where one can view their crafts^19^. Igaki et al. (1986) compared the soundness of corrosion resistance of Japanese and Indian ancient iron and found superior corrosion resistance of the Indian iron^20^. Igaki along with Prakash travelled to Loharpara, Bastar Chhattisgarh, and were able to locate the ‘Mundia’ tribes (different tribes from Agarias) and studied their iron-making technology from furnace making and process implementation’s point of view. However, detailed structure–property evaluation of the iron made by these tribes, using their traditional furnaces have not been performed^21^.

In this study, we have applied a range of advanced microscopy, spectroscopy, diffraction, and tomography techniques to unveil the hidden corrosion resistance mechanism of iron made by ancient traditional techniques in India. The reasons for the corrosion resistance of ancient Indian iron is still not clear. Different theories such as the presence of phosphorus in iron, presence of inert slag inclusions in iron, intentionally coated slag layers in iron, use of mechanical forming techniques, etc. have been proposed in different studies performed on the different ancient Indian iron artefacts. However, the corrosion resistance of iron made by tribal traditional techniques has not been examined to date to the best of our knowledge.

## Materials and methods

### Material collection

Members of the ‘Agaria’ tribes living in central India (Aamadandh, Korba (District: Korba), Chhattisgarh) donated the iron samples made through ancient Indian metallurgical techniques and using bloom furnace to D.D. for this investigation. A field survey was also conducted, and iron ore samples and slags collected from the region where the iron was made. However, the furnaces have been destroyed by the weathering action, with only a few remaining signs evident.

### Material characterization

In order to investigate the mechanism of superior corrosion resistance of iron made by tribes using ancient Indian metallurgical techniques, multiple characterization tools capable of characterizing the morphological and compositional features at various length scales (µm to nm) were used. Morphological features of the corrosion product layer formed on the top of the iron are analysed by field emission gun electron microscope (FESEM). FESEM image acquisition was conducted at 5 kV. Chemical composition of corrosion products was characterized using energy dispersive spectroscopy (EDS) and X-ray photoelectron spectroscopy (XPS). The EDS acquisition was done at 15 kV, with the EDS coupled to FESEM.

### Laboratory-based surface characterization techniques

#### Grazing incidence X-Ray diffraction (GIXRD)

We have characterized corrosion products using the GIXRD to confirm the crystalline phases present in the corrosion products. Rietveld analysis was used for evaluating the phase fraction using GIXRD pattern. The penetration depth of the X-ray is limited inside the iron sample. Therefore, X-ray can reveal the corrosion film. However, the entire iron sample could not be analysed using GIXRD. GIXRD data were collected using a PANalytical Empyrean diffractometer using Cu Kα radiation. The incident beam had a parabolic mirror with 2.3° Soller slits, and was fixed at 2° to the specimen surface. The diffracted beam had 2.3° Soller slits, a 0.18° parallel plate collimator, and a point detector with a 0.05 mm receiving slit. Diffraction data were taken over the range 10°–100° 2θ in steps of 0.02°. The diffraction data were collected in this fixed-incident beam geometry in order to enhance the contribution from surface layers. Phase identification was carried out using the software package EVA14 with the ICDD PDF2 database. Crystal structures for the phases were taken from the ICDD PDF 4 + database. Fluorescence, which is considered as an unwanted phenomena in diffraction, depends on the type of X-ray radiation source used (Copper (Cu) Kα radiation in our case) and the type of analysed material (iron sample in our case). Iron causes the development of high fluorescence with Cu Kα radiation which leads to the poor signal to noise ratio in diffraction pattern. Therefore, we have chosen neutron diffraction, used at the Australia’s Nuclear Science and Technology Organisation (ANSTO), Sydney, to analyse iron samples thoroughly because neutrons penetrate deeper inside the iron sample and provide better signal to noise ratio^22^.

#### X-ray photoelectron spectroscopy (XPS)

It is also very important to perform chemical characterization of the top layers of corrosion product film that is exposed to the atmosphere directly. Therefore, XPS was used^23^. The XPS analysis was exercised using a Kratos Axis Ultra DLD spectrometer (spatial resolution˜5 µm) with an irradiation source (operated at 225 W) of monochromatic Al Kα (1486.6 eV). The analysis chamber was kept maintained at the pressure of 9 × 10^–9^ Torr until the analysis was completed. C1s spectrum (284.8 eV) was used for calibrating the electron binding energy scale and a survey scan was carried out with the pass energy of 160 eV. CasaXPS software was used for data processing.

#### Field emission scanning electron microscope (FESEM)-EDS

The microstructural characterization of corrosion products formed on iron made through traditional ancient iron-making practices by Agaria tribes was conducted using a Field emission gun electron microscope (SEM, Zeiss NEON 40EsB).

#### Scanning transmission electron microscope (STEM)-EDS

Slag compositional analysis was conducted using FEI Titan G2 80–200 TEM/STEM at 200KV and collected high angle annular dark-field (HAADF) images along with the scanning transmission electron microscopy (STEM).

### Synchrotron and neutron based characterization techniques

#### Neutron diffraction

Neutron diffraction data were collected at the ANSTO (ECHIDNA—High resolution powder diffractometer, Sydney, Australia) over the range of 4–173° 2θ in steps of 0.5° using Ge monochromators with 0.15° mosaic spread and vertically oriented linear position sensitive detector ^3^He of 30 cm height (detector radius = 1.29 m)^24^.

#### Neutron tomography

Due to the deeper penetration depth of neutrons inside the iron sample, neutron tomography was used to extract the corrosion product layer formed on the iron as well as characterized pores’ volume inside the iron in three dimensions. Synchrotron tomography is also used for characterizing ancient iron, however, due to the development of the instrumental artefacts, we have selected the application of neutron tomography for three-dimensional characterization of iron made by tribes through ancient technology. The neutron tomographic analysis of the samples was carried out on DINGO^25^, the ANSTO imaging station (Sydney, Australia) fed by a thermal neutron beam coming from the 20 MW OPAL research reactor in Sydney. High resolution acquisition mode with an L/D ratio of 1000 was used and a field of view 55 × 55 mm^2^ was set to acquire images with a pixel size of 27 μm. A ^6^LiF/ZnS scintillation screen (thickness of 50 μm) was used. All the projections were acquired in the step of 0.25°over 360° and an exposure time of 50 s each. The tomographic reconstructions were obtained by using Octopus package^26^ while AVIZO^27^ was used for data visualization, compiling and quantification.

#### Synchrotron X-ray tomography

Synchrotron X-ray computed tomography (CT) experiment was done at Hutch 2B of the Imaging and Medical Beamline (IMBL) at the Australian Synchrotron using the ‘Ruby’ detector. It is a custom-designed detector based on a photo-sensitive device coupled by a bright lens to a suitable X-ray sensitive scintillator^28^. PCO edge sensor, which was controlled by the motorized system has been secured from direct and scatter beam radiation by a mirror. This is used to view the scintillator plate (placed orthogonally to the direction of the beam). For this experiment, the sensor was equipped with a Nikon Micro-Nikkor 105 mm/f 2.8 macro lens allowing the slide to be used as a zoom control. A terbium-doped gadolinium oxy-sulfide (Gadox, P43) scintillator 12 microns thick was used. During the experiment, the system was tuned to produce 2560 × {772}{763} pixels images giving a field of view of {33.5 × 10.1}{32.8 × 9.8}mm with a pixel size of {13.1}{12.8} microns. Data were collected using monochromatic X-rays of energies {30}{80}{120} keV, in order to ensure reasonable X-ray transmission. The pieces were positioned vertically and horizontally on the sample stage such that their centre of rotation kept the region of interest within the field of view of the detector. Each tomographic scan was collected over a 180 range in 0.10 steps, making 1800 views in total, with an exposure time of {0.07}{0.3}{1.5}s per view.

#### Small-angle neutron scattering (SANS)

SANS measurement was carried out using QUOKKA SANS instrument at the ANSTO (Sydney, Australia)^29^. Two detector positions (L = 1.3 m and L = 12 m) are used with a Q range of 0.0055715 to 0.714133 / Å. No significant neutron activation of the samples was found after the experiment^30^. SANS data were fitted using Guinier-Porod law and a combination of a Guinier-Porod law and these details can be found elsewhere^31,32^. The theory of SANS is provided in the Supplementary section.

## Results and discussion

The novelty of our study lies in determining the corrosion resistance characteristics of iron made by traditional methods developed by Indian tribes called ‘Agaria’, by using state-of-the-art analytical methods. The ancient iron was collected from the Agaria tribe members, who no longer continue the iron-making tradition. The iron sample was manufactured using ancient technology using a typical bloom furnace. Construction of this type of furnace is well described in the article published by Juleff et al. (1996)^2^. Metal products from an ancient bloom furnace are very rare to find and pieces of evidence of furnaces have been destroyed due to weathering actions. It has been suggested in the literature that Agaria tribe’s ancient iron-making technology using bloom furnace was in place even before 1200 AD, but the exact date is not available^33^.

The initial step in this study involved collection of the iron samples from tribes (Suppl. Figure 1 (a) and (b)) and iron ores which were used in making ancient irons by tribes from the forest of Aamadandh, Korba, Chhattisgarh, India.

**Figure 1:**
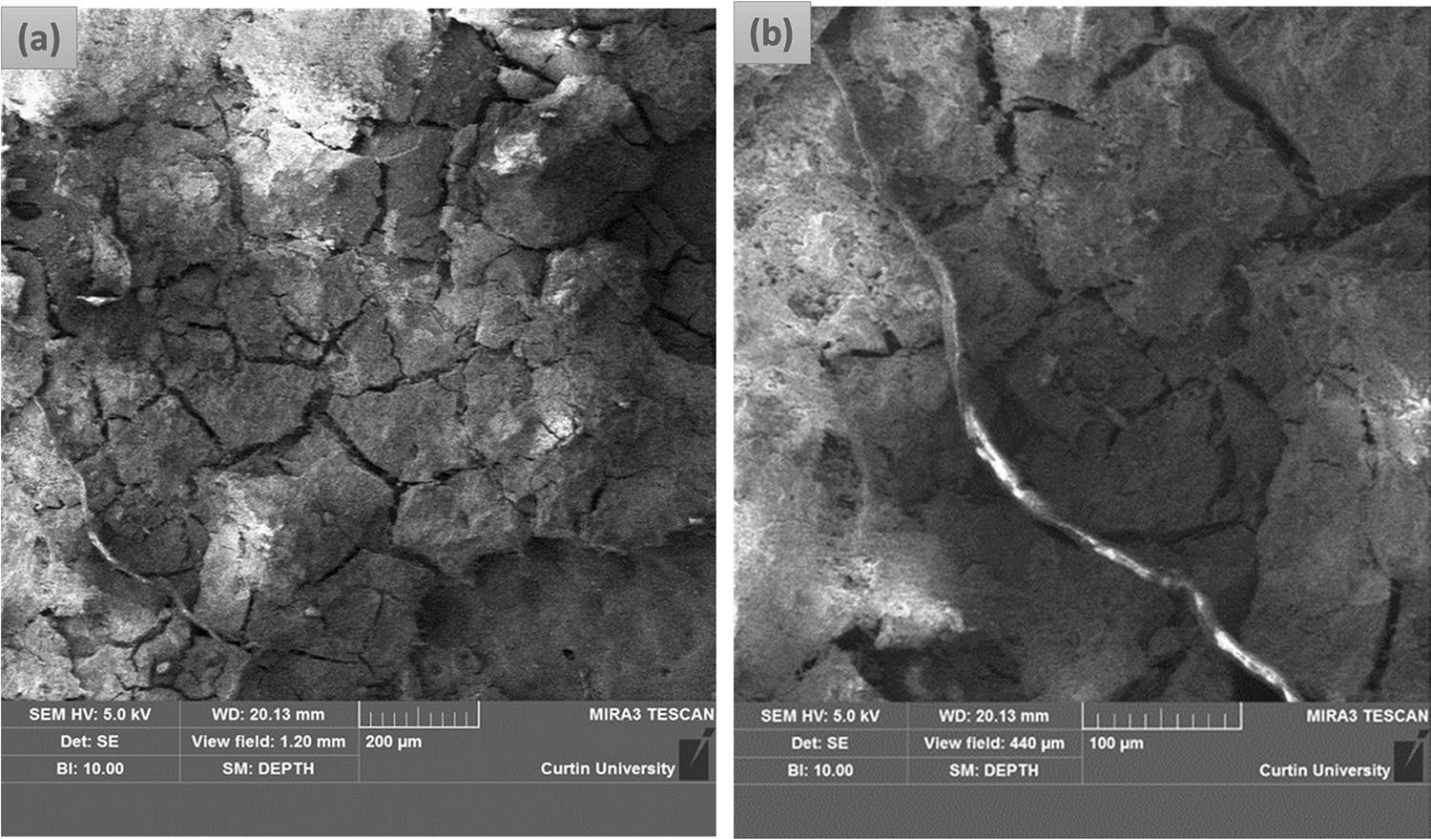
(**a**) FESEM images of (**a**) the morphology of the corrosion product film formed on the surface of iron made through ancient Indian metallurgical methods; (**b**) flake formation in the film.

We have collected and analysed ore samples from the same region (see Suppl. Figure 1 (c)). In the iron making process, these ores were placed inside the bloom furnace. Bowl-shape bloom furnaces were usually built below the ground level by digging a pit (typically of cylindrical shape) with dimensions of about 800 mm in height and 200 mm in diameter. The shaft of the furnace was constructed below the 600 mm mark. The construction design of the furnace was completed by a bowl-shaped hearth with a typical diameter of about 240 mm and a depth of about 100 mm. The bowl-shaped hearth had a hole for tapping out slag (i.e. removing slag from the furnace), which is the waste product of the iron making process. Images of final iron products and slags (by Agaria tribe’s traditional iron-making process) collected from the site are shown in Fig. 1 (a). The placement of the furnace below the ground level was designed to reduce the excess air inside the furnace. It must be highlighted that air blowing was used to maintain the temperature at around 1150 °C. Iron-making was designed to take around 5–6 h/kg iron production and semi-fused mass of sponge iron block came out at the end with a significant amount of slag. The slags that remained in the final iron block were removed using forging technique^21^.

Morphological analysis of corrosion product layer of the iron sample (see Fig. 1 (a)) was conducted using field-emission scanning electron microscope (FESEM). Figure 1 (a) reveals the formation of a thick corrosion product layer on the top of the iron with obvious cracks. It is evinced from Fig. 1a that cracks were in the order of micrometres in size and the majority of the cracks are in the range of ~ 4–5 µm, and a few large cracks were also observed. At the surface areas with a thicker surface film, less cracks occurred. Flake formation is noticed in the FESEM image (Fig. 1b) which is a common characteristic of atmospheric corrosion. It is evident from earlier studies that fine flakes are the characteristic for atmospheric corrosion process whereas coarse flakes resemble marine corrosion^34^, which is clearly evident from Fig. 1b.

**Figure 2:**
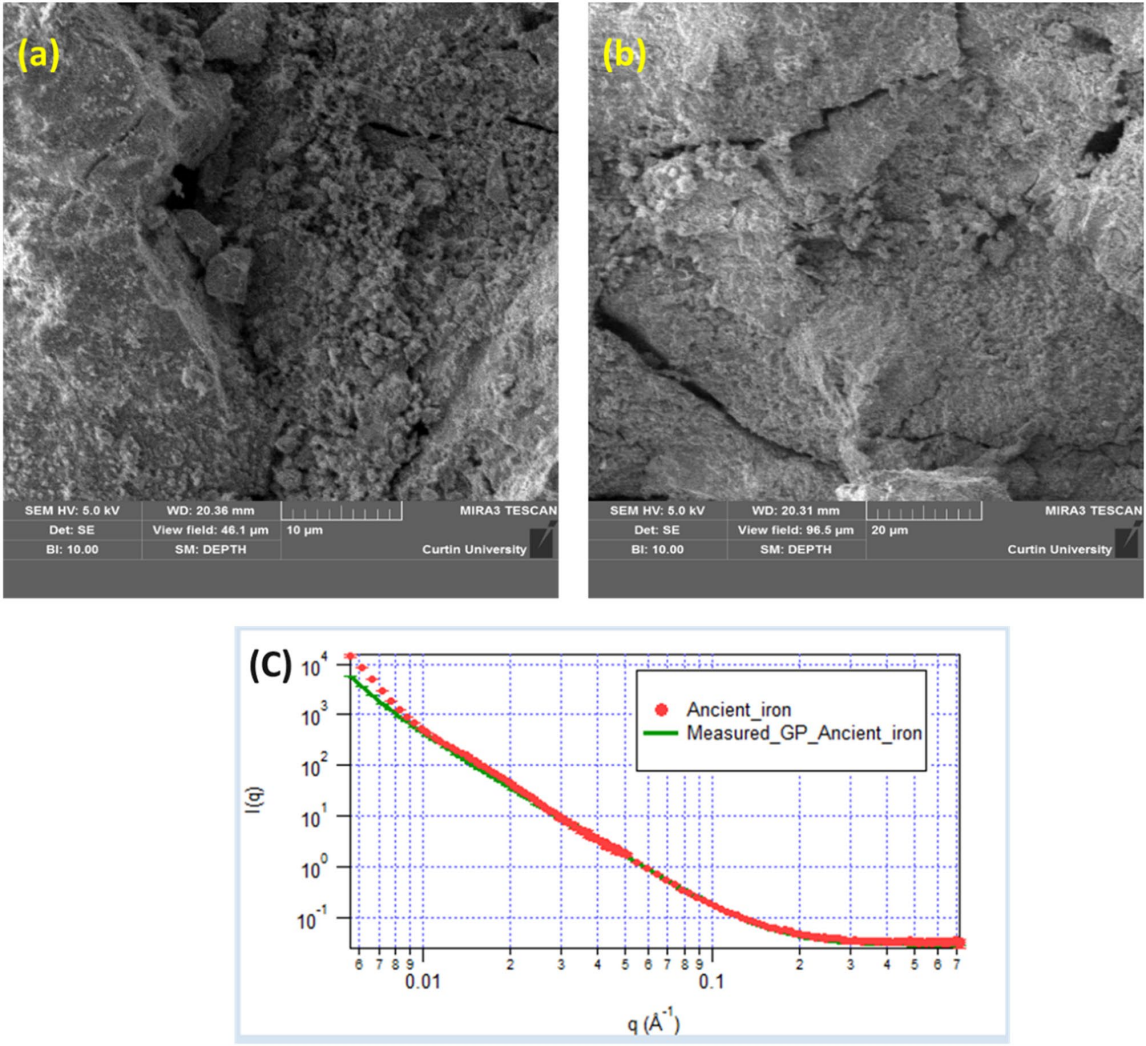
(**a**) FESEM image of the corrosion product film found on the top of the iron made by Agaria tribe (**b**) FESEM image depicting the inhomogeneity in the shape and size of the corrosion phase particles that are found in corrosion product film (**c**) represent the small-angle neutron scattering (SANS) scattering data collected from the iron made through ancient Indian metallurgical methods, scattering data is fitted with the Guinier-Porod model.

The corrosion product film is known to be composed of small-size particles of different phases, as evident later in this study. We anticipate that the particle-precipitation phenomenon led to formation of the phases through nucleation and growth that are found in corrosion product film^34^. Nucleation of the initial phase (of corrosion product) is known to assist in the nucleation of another phase by acting as a seed. We believe that phenomenon would have led to the formation of a thick corrosion layer^35^. Cox et al. (1994) suggested the importance of pre-existing oxide layer for nucleation of secondary phase particles or new oxide phases during the formation of passivation layer^35^. Therefore, we hypothesize that the nucleation and growth of corrosion product layer are also supported by slag phase particles present in the iron. We found that it is difficult to predict the exact phenomenon of growth of corrosion product film formation in the uncontrolled corrosion experiments (like in this study) because the nucleation and growth is a complex phenomenon and depends on various factors such as substrate crystallographic nature, interface between the substrate and layer, specific free surface energy, adhesion energy, etc^36^. Corrosion product film is found composed of random shape particles as depicted in FESEM analysis (Fig. 2a,b). Mild heterogeneities in morphological features confirmed the less aggressive atmospheric corrosion^34^.

**Figure 3:**
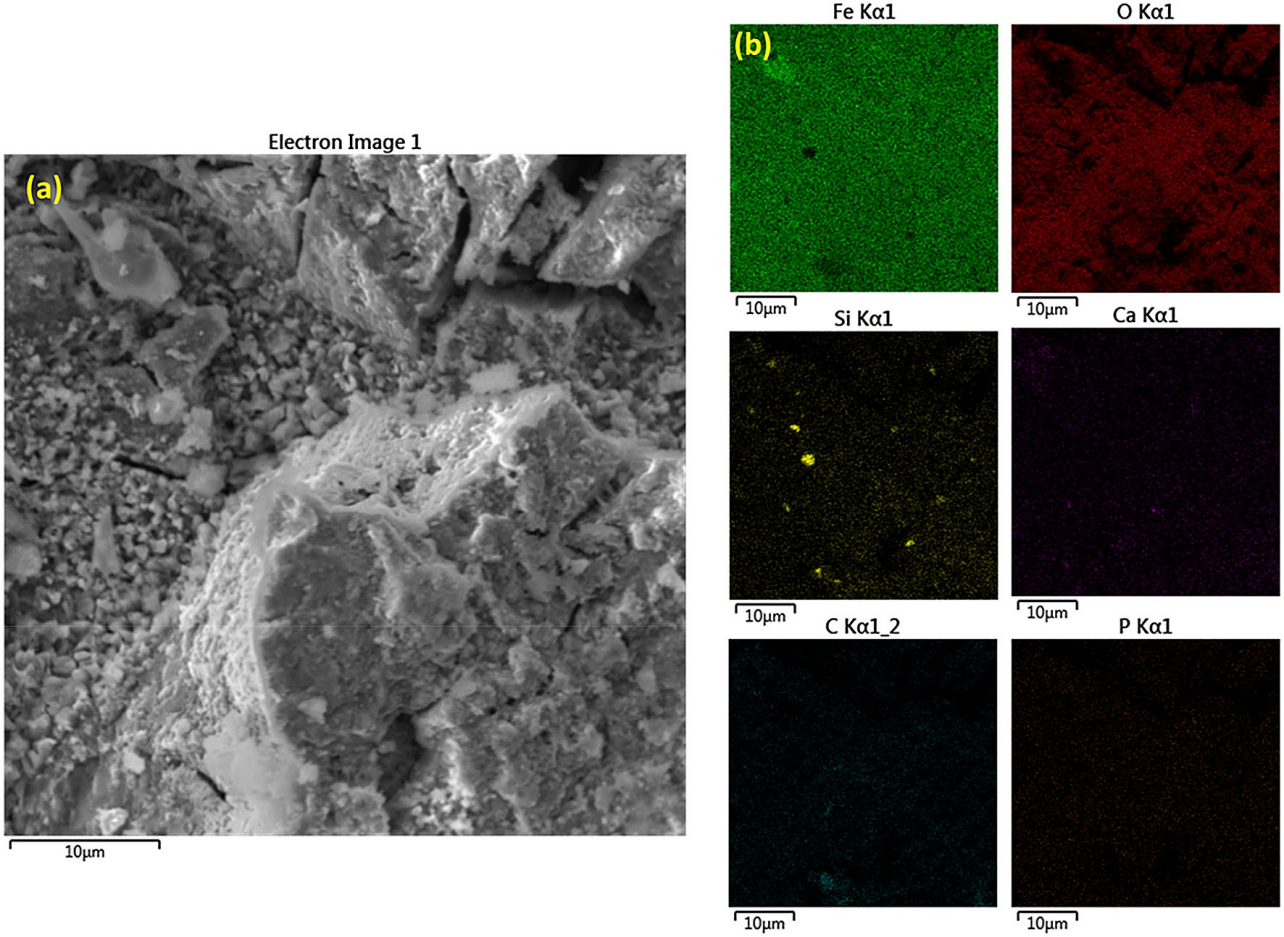
(**a**) FESEM images of corrosion product film formed on the surface of iron made through ancient Indian metallurgical methods (**b**) EDS mapping of Fe, O, C, Si, Ca without P (dots appeared in P mapping are due to instrument noise).

Further features of corrosion product film was characterised using small-angle neutron scattering (SANS) (Fig. 2c). Scattering data were fitted with a shape-independent model called Guinier-Porod model^31^ which revealed the formation of a rough surface with an interlinked structure on the top of the iron, with a Porod exponent of m = 3 (Fig. 2c). Additionally, shape-independent model fitting was performed to investigate the surface features of the corrosion product film using SANS. These results are well-corroborated with the FESEM analysis which exhibited the formation of a rough heterogeneous corrosion product film. The compositional analysis of the corrosion product film is performed by FESEM-energy dispersive spectroscopy (FESEM-EDS) which confirmed the presence of Fe, C, O, Si and Ca (Fig. 3a,b-EDS mapping).

**Figure 4:**
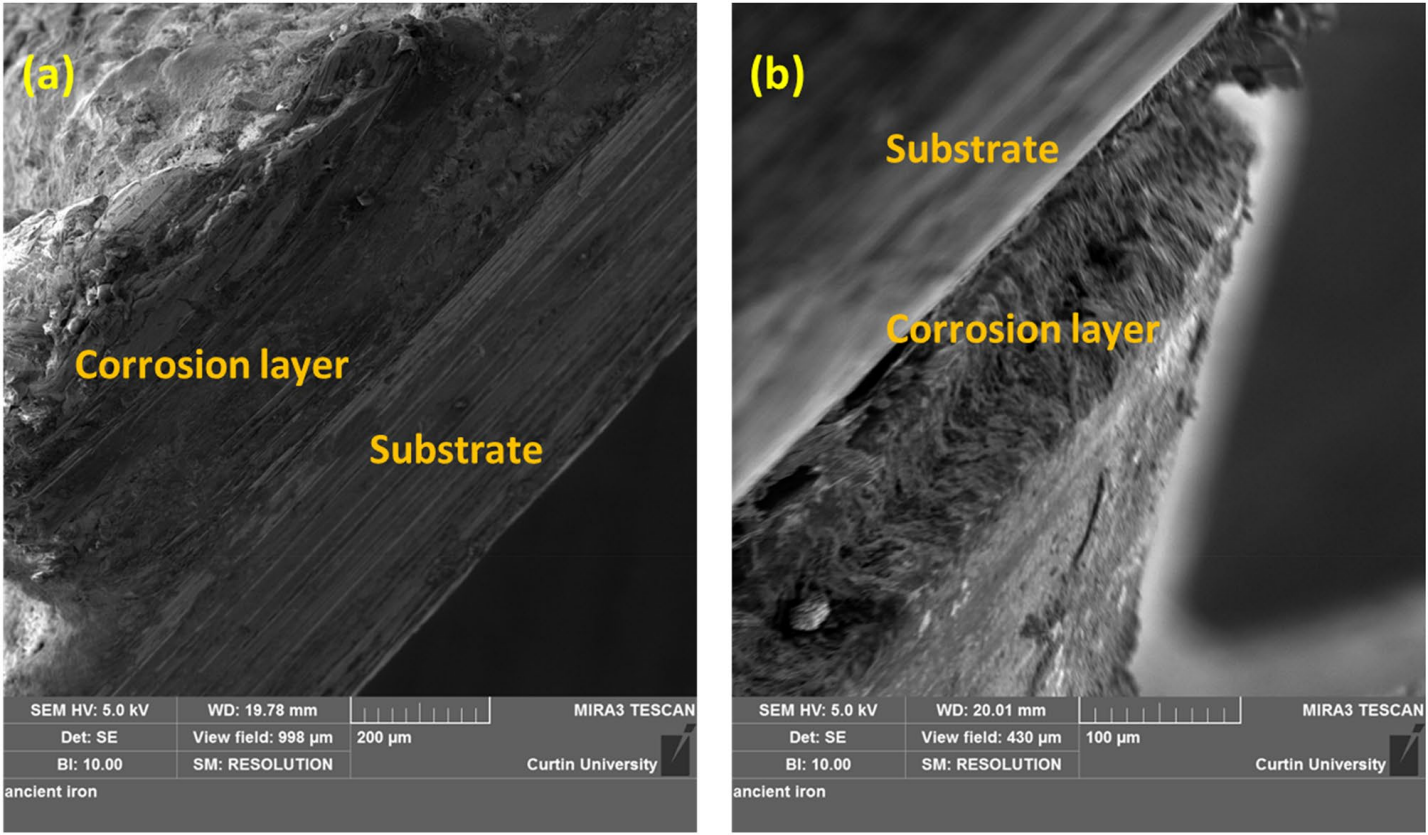
Cross-sectional FESEM images of corrosion product film formed on iron made through ancient Indian metallurgical methods depicting (**a**) interface formation between corrosion product film and substrate (iron) (**b**) interface formation between corrosion product film and substrate (iron) at different location and in different (view) orientation depicting the small holes on film.

Earlier proposed theories discard the utilization of limestone in ancient Indian iron-making furnaces because of the absence of CaO in slag which led to the high P retainment in iron^37^. On the contrary, the presence of P and Ca is confirmed by the STEM-EDS analysis of collected slags (Suppl. Figure 2). It is worth mentioning that the removal of P from the iron (to slag) depends on various factors, such as the presence of CaO and other basic compounds. This theory is well corroborated with the principles of slag thermodynamics and chemistry that suggest higher P removal with CaO than the FeO^38^. It is noteworthy that the P can lead to the formation of liquid phosphides at the grain boundaries and cause cracking during mechanical working such as forging^39,40^. Therefore, in order to clarify further that whether CaO was added intentionally during the iron making process to remove P from iron, we have characterized the iron ores, which were used in iron making practice and collected from the same site, using XRD. The XRD analysis (Suppl. Figure 3) confirms the presence of hematite, kaolinite, and anatase phases and excludes the presence of Ca in the ores. We speculate that the addition of Ca to the iron was through the clay used for preparing the bottom part of the furnace along with the fine coal dust. It is worth mentioning that a slanting platform made from clay-coated bamboo was used for sliding the charge into the furnace and therefore some Ca addition is also possible from this operation. However, the probability of introducing Ca into the melt from the bottom portion of the furnace is more likely compared to the slanting platform used for the charge sliding^19^. Figure 4 and Suppl. Figure 4 elucidate the outwards segregated diffusion of slag constituents such as Si and Ca. (Slag constituents are expected to come from the iron ore used in the iron-making by tribes as discussed later with the help of XRD of iron ore analysis, which confirms the presence of kaolinite.) This phenomenon led to the localized dissolution of Fe in the corrosion layer. Metal dissolution is considered as anodic reaction whereas oxygen reduction is known as a cathodic reaction (see the reaction (1)). Significant depletion of Fe is observed which affirmed the anodic dissolution. This depletion of Fe causes the diffusion of Si and Ca that were found segregated at few locations. It is also noteworthy that the diffusion of Si and Ca from the surface of iron into the corrosion layer (where particularly Fe was found depleted) is attributed to the raising oxygen potential due to the combined effect of formation of FeO layer and electrochemical erosion^41^. Buffered surface of iron discards the hydrogen reduction as prominent cathodic reaction such as:

1 $${\text{2Fe }} + {\text{ H}}_{{2}} {\text{O }} + \frac{3}{2}{\text{O}}_{{2}} = {\text{2FeOOH}}$$

It is also noteworthy that initially formed lepidocrocite is further transformed to goethite and spinel iron oxide-hydroxide. This continuous transformation resulted in the formation of maghemite and hematite (as observed). Hematite is the most stable phase among these two phases due to highly negative Gibbs free energy^42^. FESEM-EDS mapping (see Fig. 3 and Suppl. Figure 4) showed the formation of a slag layer consisting of Ca and Si with Fe and O in the corrosion layer. It was well documented in earlier research that ion-exchange driven non-stoichiometric removal of cations drives the inward diffusion of hydrogen species (H_3_O^+^, H_2_O)^43^. Atmospheric corrosion can lead to this condition that offers outward diffusion of cations, while alkali ions in this study are either dissolved or diffused inwards. We, therefore, speculate that Fe acted as an anodic cell due to the formation of a localized galvanic cell composed of Fe as an anode, whereas slag phases acted as the cathode and appeared to be dissolved at certain areas of the corrosion layer. This localized chemical imbalance led to changes in the chemical potential which drove the diffusion of Si and Ca and assisted in their segregation^43^.

The presence of slag phases in the corrosion layer of iron was further confirmed from the EDS spectra from various locations at the surface (see Suppl. Figure 4). Ca, Si, Al were detected along with Fe, C, and O. Localized dissolution of Fe and non-homogenous distribution of slag elements were further noticed from the EDS data, confirming our earlier argument of localized cell formation that leads to diffusion of ions. Formation of bubbles in the iron was also noticed, which is a typical characteristic of iron made in bloom furnace^44^. EDS analysis confirms the presence of Fe, O, Si, Al, Ca, and Ti; without any presence of P. It is speculated that the surface segregation of Ti would have played an important role in making the Agaria tribe’s iron corrosion resistance. A recent study conducted with TiO_2_-clay composite also confirmed the hydrophobic nature of this composite^45^. Therefore, we hypothesize that the surface segregation of Ti would have supported in the formation of the protective passive-film on iron and helped Agaria tribe’s iron to be corrosion resistant. The source of Ti addition in iron was iron ores which were used in the iron-making process and contain 4 wt% of ilmenite (see Suppl. Fig. 3). Therefore, it is believed that Ti was not alloyed in iron intentionally. We have carried out STEM-EDS (elemental mapping) (see Suppl. Figure 2) of slag, which is a by-product of the iron-making process. Ti was not evidenced from the STEM-EDS mapping. Hence, it is believed that Ti was added in the iron material, namely from iron ore with anatase that was used in the iron-making process by tribes. The role of Ti in the prevention of metal corrosion is well described^46^.

In order to investigate the interface between the iron and the corrosion film, cross-sectional FESEM was performed (see Fig. 4a,b) and shows the formation of a coherent interface with a thick corrosion layer.

Cross-sectional FESEM images confirmed (within the resolution limit of FESEM) the absence of pores and pits at the interface and showed coherent interface as one of the paramount factors responsible for high corrosion resistance of the ancient iron. It is worth mentioning that defects such as holes were noticed on the top of the corrosion product layers. However, we could not identify from the FESEM data whether these holes extended to the iron surface. We have therefore employed neutron tomography as mentioned later.

Correlative microscopic and spectroscopic techniques are found useful for in-depth analysis of corrosion products formed on the ancient iron artefacts^47,48^. In this study, corrosion layer was analyzed using GIXRD. The formation of hematite was exhibited in the diffraction pattern obtained by GIXRD analysis (Suppl. Figure 5). GIXRD analysis of corrosion layer has confirmed the presence of hematite (Fe_2_O_3_), quartz (SiO_2_) and calcite (CaCO_3_), while Rietveld analysis proved the formation of Fe_2_O_3_ (70wt%) along with SiO_2_ (19wt%) and CaCO_3_ (11wt%) (Suppl. Figure 5). These protective corrosion products might have led to high corrosion resistance^47^. However, the signal to noise ratio was poor and the ancient iron sample was thus further characterized using neutron diffraction. In fact, neutrons are electrically neutral and can penetrate deeper inside the ancient iron. Neutron diffraction confirmed the presence of iron (Fe), cementite (Fe_3_C, cohenite in mineralogy) and maghemite (ϒ-Fe_3_O_4_), but a few diffraction peaks remained unclassified. Rietveld analysis of neutron diffraction pattern confirmed the association of ~ 92 wt% of Fe with 1.1 wt% of Fe_3_O_4_ and 1.7 wt% of Fe_3_C (Suppl. Figure 6). Unidentified phases were about 5 wt%. The unidentified peak positions (at 2θ°) were 40.62°, 42.38°, 64.49°, 76.86°, 96.73°, and 115.34°. Neutron diffraction data confirmed the formation of BCC α-Fe. Detection of BCC α-Fe made us believe that Agaria’s furnace was suitable to be operated below 1000 °C.

**Figure 5:**
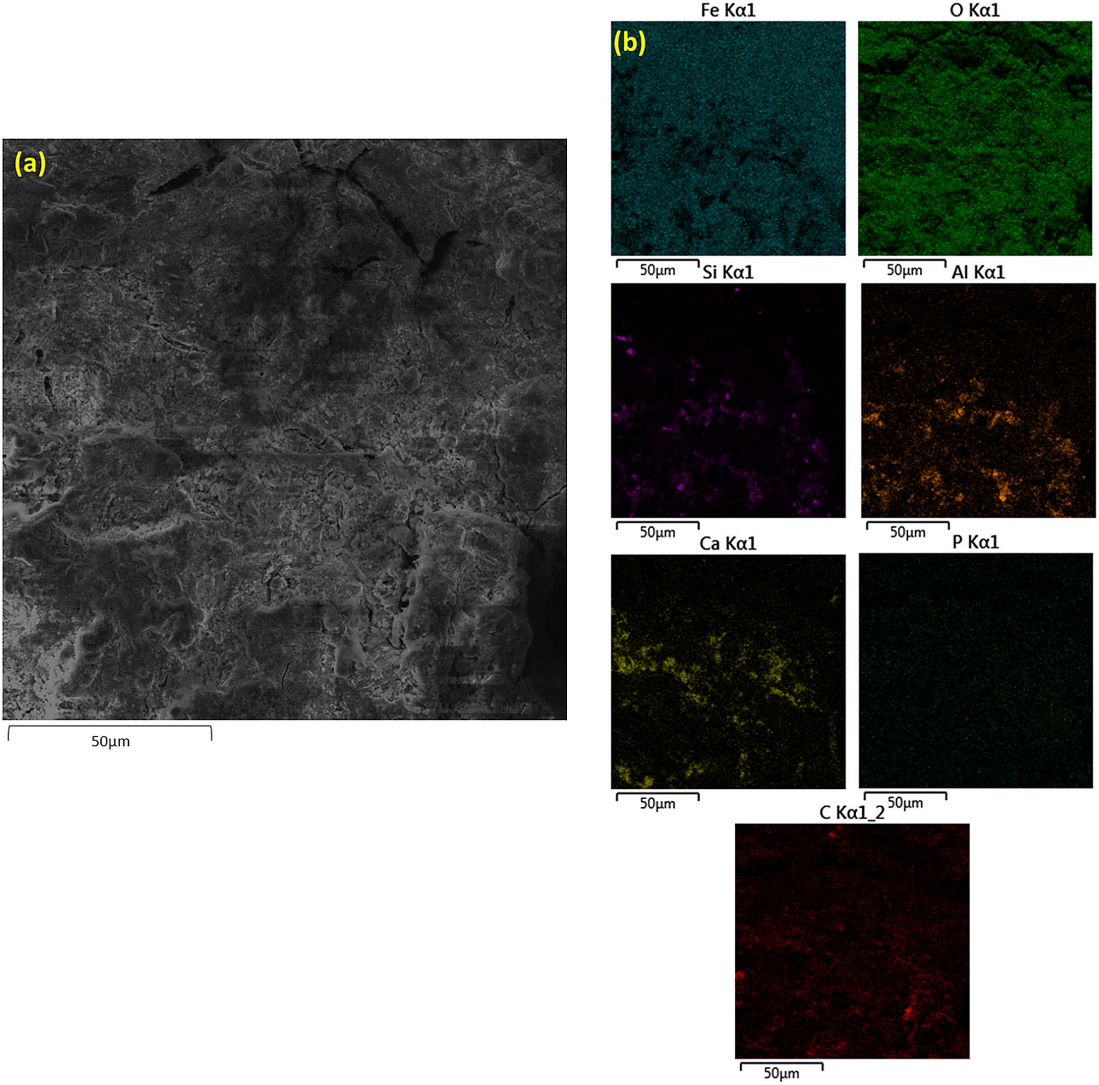
(**a**) FESEM images of corrosion product film formed on the surface of iron made through ancient Indian metallurgical methods exhibits (**b**) EDS mapping of Fe, O, Si, P (dot points indicate the instrument noise), Ca, Al and, C.

**Figure 6:**
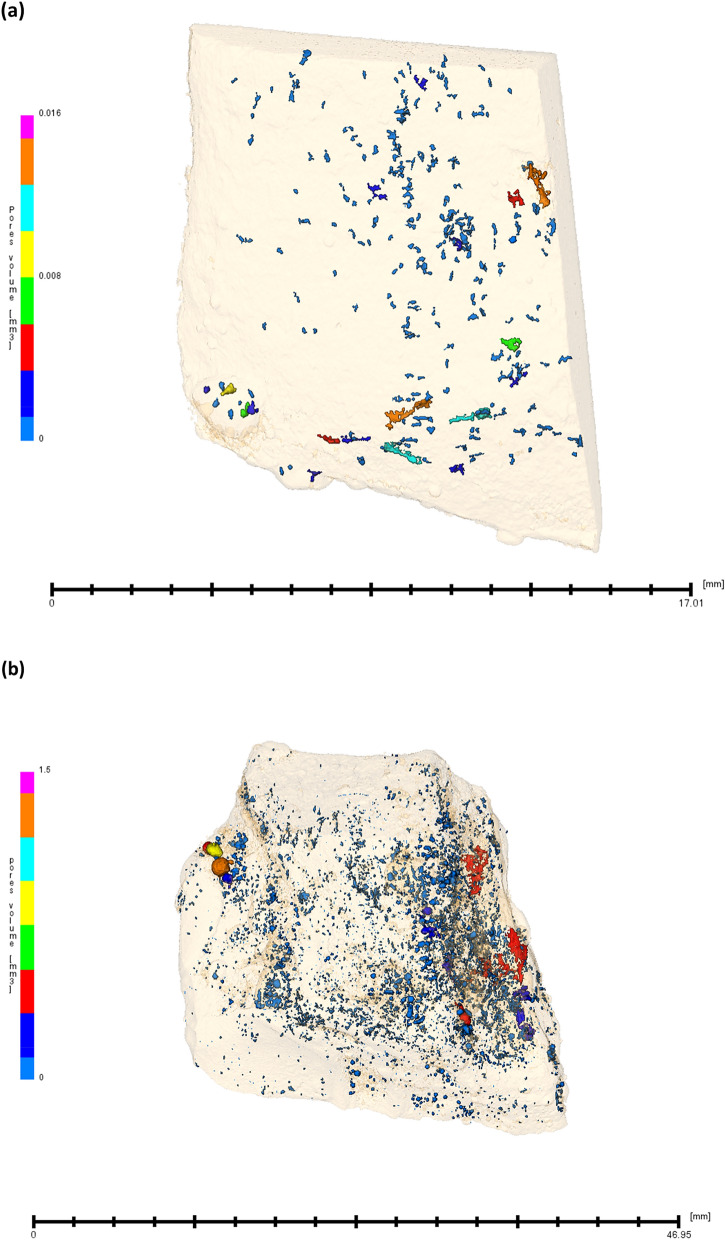
Neutron tomographic images depicting the volume of the pores that are formed on the iron made through ancient Indian metallurgical methods in (**a**) un-hammered iron (**b**) hammered (forged) iron.

The corrosion product film was found composed of hematite, maghemite and slag phases as confirmed by GIXRD and neutron diffraction. Maghemite is expected to be formed underneath these layers as detected in neutron diffraction analysis (Suppl. Figure 6), which is a technique used for bulk-material characterization. Among these iron oxide polymorphs, hematite is the most stable iron oxide with Gibbs free energy of formation of –744.4 ± 1.3 kJ mol^-1^ whereas maghemite is considered a less stable polymorph with Gibbs free energy of formation of –731.4 ± 2.0 kJ mol^-1^ at 298 K and 1 bar pressure^49^. It is believed that the formation of oxides could have occurred through internal oxidation mechanism^50^. Internal oxidation allows oxygen to enter into the material and this diffusion process leads to the sub-surface precipitation of oxides of alloying elements. The concentration of these solute elements is imperative and decides the transition between internal to external oxidation. We have noticed segregated Si in EDS mapping, which indicates the long-range diffusion of Si from the matrix (parent metal). It is also worth to note that this diffusion makes the chemical potential of Si (in the oxide zone) lower than the surrounding matrix and is considered the driving force for the Si diffusion. However, in EDS mapping analysis, we have noticed the widespread Si and Ca with O which confirms the diffusion of these elements from the grain/sub-grain of the matrix (iron) to the oxygen diffusion zone. We have confirmed the formation of several oxide zones (multilayer oxide formation), which is a typical characteristic of internal oxidation, by using various techniques (GIXRD, neutron diffraction, XPS, synchrotron and neutron tomography). Hematite is the most stable phase among the iron oxides^49^ and maghemite is therefore expected to be transformed into hematite thermodynamically. During the initial stage of oxidation of Fe, outward diffusion of Fe leads to the vacancy formation which later accumulates as cavities. Cavities are responsible for the formation of micro-channels that are known to pave the path for fast oxidation^51^. Corrosion film is found with cracks which seem to be formed during the oxide scale growth on Agaria tribe’s-iron as illustrated by Neff et al. (2005)^47^. These cracks are expected to provide a path for continuous oxidation of Fe till the passive calcite and quartz phases are formed. It is noteworthy that precipitate phase formation depends on the localized presence of ions (e.g. Ca^2+^, CO_3_^2-^, etc.)^47^. However, the stability of these phases depends vastly on the atmosphere^52^.

Earlier studies have delineated the formation of crystalline iron hydrogen phosphate hydrate (FePO_4_·H_3_PO_4_·4H_2_O), α-, γ-, δ-FeOOH and magnetite in the case of Delhi iron pillar whereas Gupta dynasty’s Eran iron^37,53^ has shown the presence of γ -FeOOH and δ –FeOOH. It is worth mentioning that the researchers were unable to detect phosphate phase (formed due to the phenomenon called micro-segregation) using Mössbauer spectroscopy which was identified using micro-XRD^54,55^. In this study, the presence of hematite and maghemite were identified as scale phases on the top of the iron made by the Agaria tribe. P is found present in slag whereas the presence of P in iron was not detected within the limit of the analytical techniques used in this study. On the basis of this result, we speculate application of lime and other basic compounds during the iron making process which would have led to the transfer P to slag.

The presence of slag elements such as Si, Ca (along with the Fe and O) in the scale grown on iron made by Agaria tribes is confirmed by XPS (Suppl. Figure 7). Oxides of these elements (Fe, Si and Ca) are generally known to be cathodic in nature and do not allow current to flow through and help in protecting iron from corrosion. These observations depict cathodic prevention as one of the reasons for the high corrosion resistance of the iron made through ancient Indian traditional methods. Second phase particles (such as slags and unreduced oxides) act as cathodic sites whereas metals such as Cr and Mn in the pure iron act as anodic sites. Second phase particles are known to form passive layers^16^.

**Figure 7:**
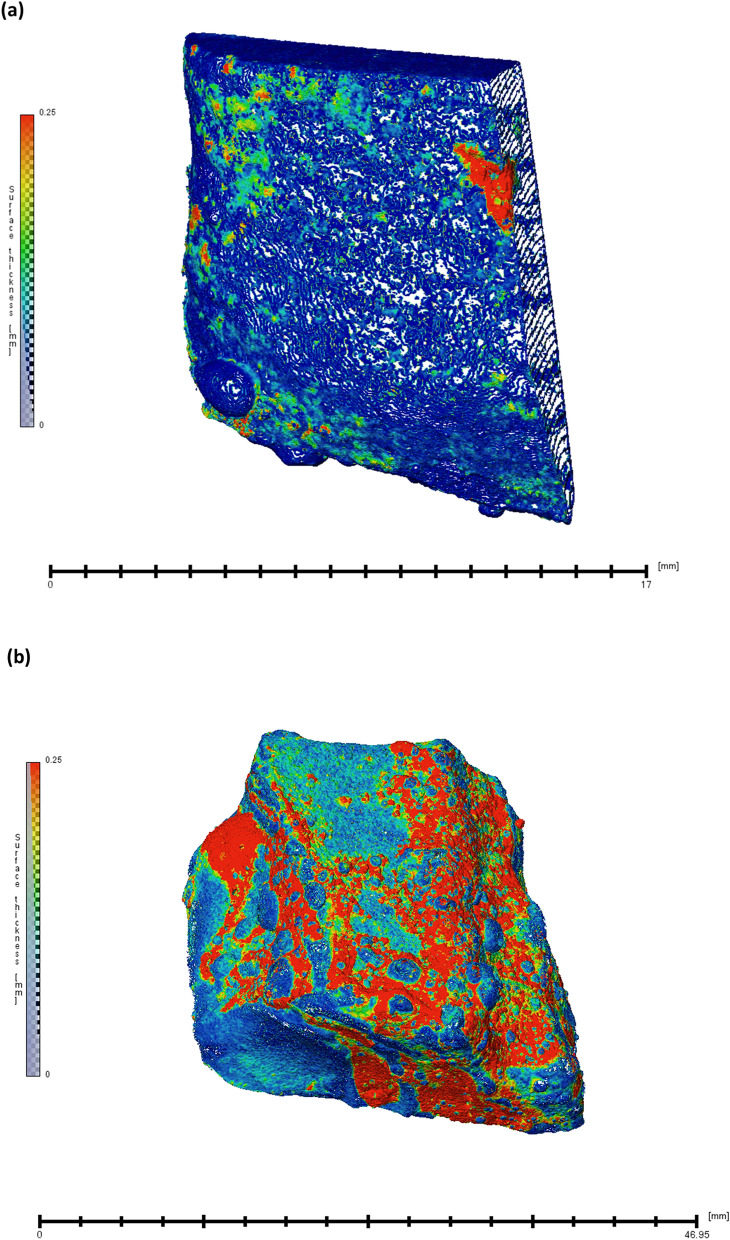
Neutron tomographic images of corrosion product film thickness that are formed on the iron made through ancient Indian metallurgical methods in (**a**) un-hammered iron (**b**) hammered (forged) iron.

The XPS survey scan does not show the presence of Al, which was noticed in FESEM-EDS (Suppl. Figure 4). This postulates that the Al-containing slag phase might have formed close to these layers and was beyond the resolution of the XPS instrument. This also means that the slag phases were formed at different depths within the corrosion scale formed on iron. It has been postulated earlier that mobile oxygen ions lead to the formation of a slag-oxide interface with the formation of large amounts of iron oxide products. In this study, iron corrosion products along with the slag phases are detected in the film formed on the top of the iron. We therefore speculate that atmospheric O played an important role in building up the inhibitive layers on the iron surface. It is observed from the FESEM-EDS mapping figures (Fig. 5a,b) that Si, Ca, and Al—slag phase elements are not segregated but randomly distributed and can be therefore considered as mobile elements at the surface of the corrosion layer. Also, Fe was found depleted in a few instances.

Similarly, these slag elements also remained non-depleted which indicates formation of microscale galvanic cells in the corrosion product layer. This highlights the slag-phases’ role in maintaining the cathodic passivity that provides corrosion protection to the underlying steel^56^.

We have also analysed the un-hammered and hammered iron with the help of neutron tomography (Fig. 6a,b) (see suppl. Figure 8 for the 3-D projection of different orientation views), which confirmed the presence of small volume pores. Hammering (forging) is known to remove the impurities (such as slag, pores, etc.) and to consolidate the pores available in materials. The consolidation of pores in hammered iron can also be seen (Fig. 6b). The values of the pores’ area (in 3-D) is found consolidated and most of them are found in the region of less than 1mm^2^ in hammered iron, whereas un-hammered iron has exhibited the formation of large size pores, mostly found concentred within the region of 1 to ~ 3.5mm^2^ (see Suppl. Figure 9 (a)). The majority of pores’ volume that is depicted in un-hammered and hammered iron are found below ~ 0.002 mm^3^ and below ~ 0.04 mm^3^ respectively (see Suppl. Figure 9(b)). The mathematical value of the pores’ volume in hammered iron is found higher than the un-hammered iron. On the other hand, the quantities of the pores inside the iron are also found higher in hammered iron. We speculated that this phenomenon could be attributed to the dislodgement of the inclusions (detachment of slag phases) from iron during the hammering (forging) operation. However, the superlative corrosion resistance of hammered iron could be associated with the thick passive corrosion product film formation on the top of the iron as delineated earlier in this article which made iron remained inert. It is also noteworthy that the porosity obtained in neutron tomography was present inside the iron. A comparison of histograms of equi-diameters and length of the pores are shown in Suppl. Figure 10 (a) and (b). We also analysed the pores present in iron made by Agaria tribes using synchrotron X-ray tomography. However, the low transmission and scattering through the sample material altered the quality of the data by introducing strong artefacts and prevented accurate porosities analysis. On the other hand, the neutron tomography images were free from these artefacts (See Suppl. Figure 11).

In order to further relate the high corrosion resistance to the presence of a protective surface film, we have carried out the analysis of the corrosion product film thickness at the hot hammered (forged) and un-hammered (un-forged) iron. It is noticed that the thicker corrosion product film was present on the hammered iron compared to the un-hammered iron. As illustrated earlier, metal-working process (mechanical metallurgical operations) called hot hammering (forging) is known for removing impurities such as slags and consolidating internal pores, we hypothesize that the metal-working operation carried out by Agaria tribes has a great impact on making the iron corrosion resistance. We hypothesize that thick corrosion product layer, which is known to protect the substrate from further corrosion, formed on hammered iron and consolidated pores formation can play a vital role in iron’s corrosion resistance (See Fig. 7 (a) and (b); and Suppl. Figure 12).

## Conclusions

In summary, we have investigated the iron manufactured using the traditional iron making process followed by Indian tribes known as ‘Agaria’. Our results clearly demonstrated the importance of analytical techniques operating at various length scale, in proposing the mechanisms of corrosion resistance including other properties of archaeomaterials. We have proposed the hypothesis for the excellent corrosion resistance of Agaria-tribe’s iron. In this article, the role of iron ore’s composition in the formation of corrosion products film (protective film against corrosion) on the iron surface is highlighted. Hematite and maghemite along with slag phases were depicted by using GIXRD and neutron diffraction as the corrosion products on the iron surface. The investigations also highlighted the essential role of a multi-analytical techniques approach in the analysis of archaeomaterials. These findings were identified as the primary reasons for the high corrosion resistance of the iron. Unlike Delhi iron pillar, P (which was found in the iron of Gupta’s period) is not found in the corrosion product layer formed on the iron (irrespective of hammered/un-hammered) manufactured by Agaria tribes within the limit of the correlative analytical techniques that are used in this study. However, the scope of further investigation still prevails. The effect of metal-working operation (followed by Agaria tribes) in making the iron corrosion resistance is also demonstrated. We hypothesize that the metal working operation known as hot hammering, has led to the consolidation of internal pores (which were present in iron before pre-treatment) and removal of inclusions such as slag. Thicker passive corrosion product film, which is known to be protective on the Agaria tribes’ iron, is observed for hammered iron than un-hammered iron. Hence, it is postulated that consolidation of pores, expected removal of inclusions and thick passive corrosion product layer formation have supported the iron in attaining the excellent corrosion resistance. We anticipate that our findings will assist in uncovering the hidden knowledge pertaining to the archeomaterials’ (ferrous) degradation phenomenon, particularly in Japan and the South Asian region’s, with a history of close collaboration in ferrous metallurgy.

## Supplementary Information

Supplementary Information.
