# Supplementary material for: Uncovering the superior corrosion resistance of iron made via ancient Indian iron-making practice
Source: Sci Rep. 2021 Feb 19;11:4221. doi: 10.1038/s41598-021-81918-w (PMC7896077; doi:10.1038/s41598-021-81918-w)
Supplement: Supplementary file 1 — Supplementary Information. [file 41598_2021_81918_MOESM1_ESM.docx]

**Supplementary Information**

**Uncovering the superior corrosion resistance of iron made via ancient Indian iron-making practice**

*Deepak Dwivedi^1^, Jitendra P. Mata^2^, Filomena Salvemini^2^, Matthew R. Rowles^3^, Thomas Becker^4^, Kateřina Lepková^1^*^*^

*^1^Curtin Corrosion Centre, WA School of Mines: Minerals, Energy and Chemical Engineering, Faculty of Science and Engineering, Curtin University, Australia*

*^2^Australian Centre for Neutron Scattering, Australian Nuclear Science and Technology Organisation (ANSTO), Lucas Heights, NSW-2234, Australia*

*^3^John de Laeter Centre, Curtin University, Perth, Australia*

*^4^School of Molecular and Life Sciences (Chemistry), Curtin Institute for Functional Molecules and Interfaces, Faculty of Science and Engineering, Curtin University, Australia*

*Correspondence to: [K.Lepkova@curtin.edu.au](mailto:K.Lepkova@curtin.edu.au)


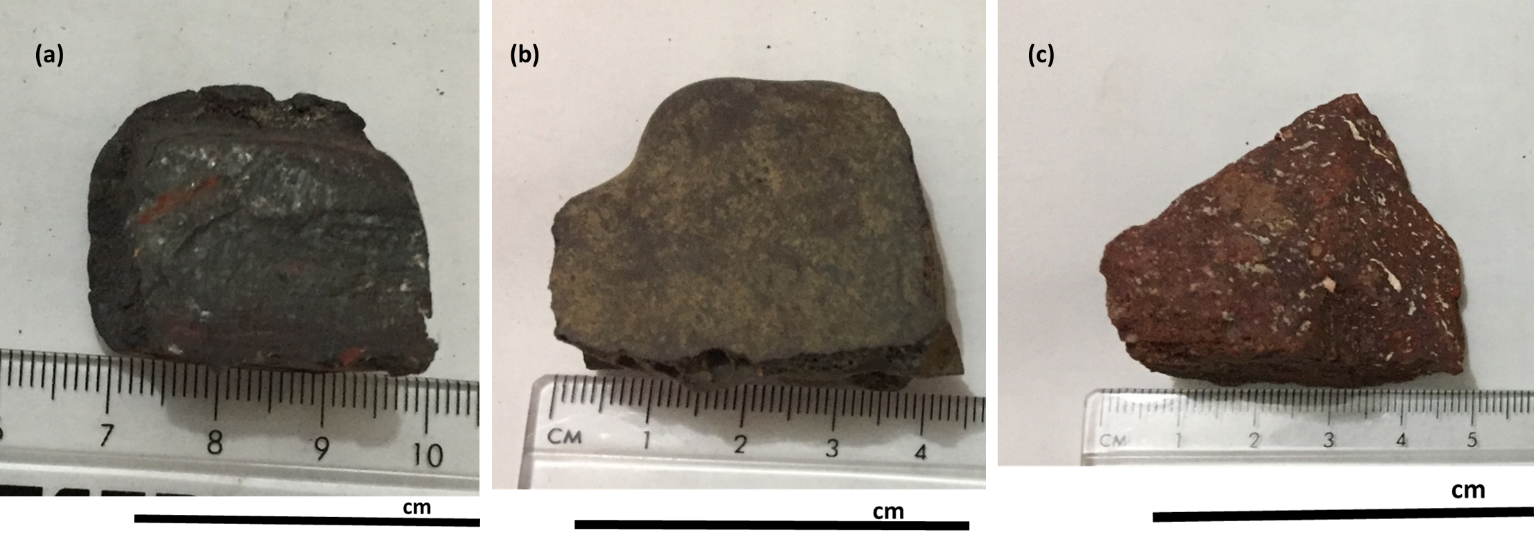


**Suppl. Figure 1**: Photographic images of (a) iron made through ancient Indian metallurgical method followed by Agaria tribes (b) slag sample (c) iron ore used in iron-making.


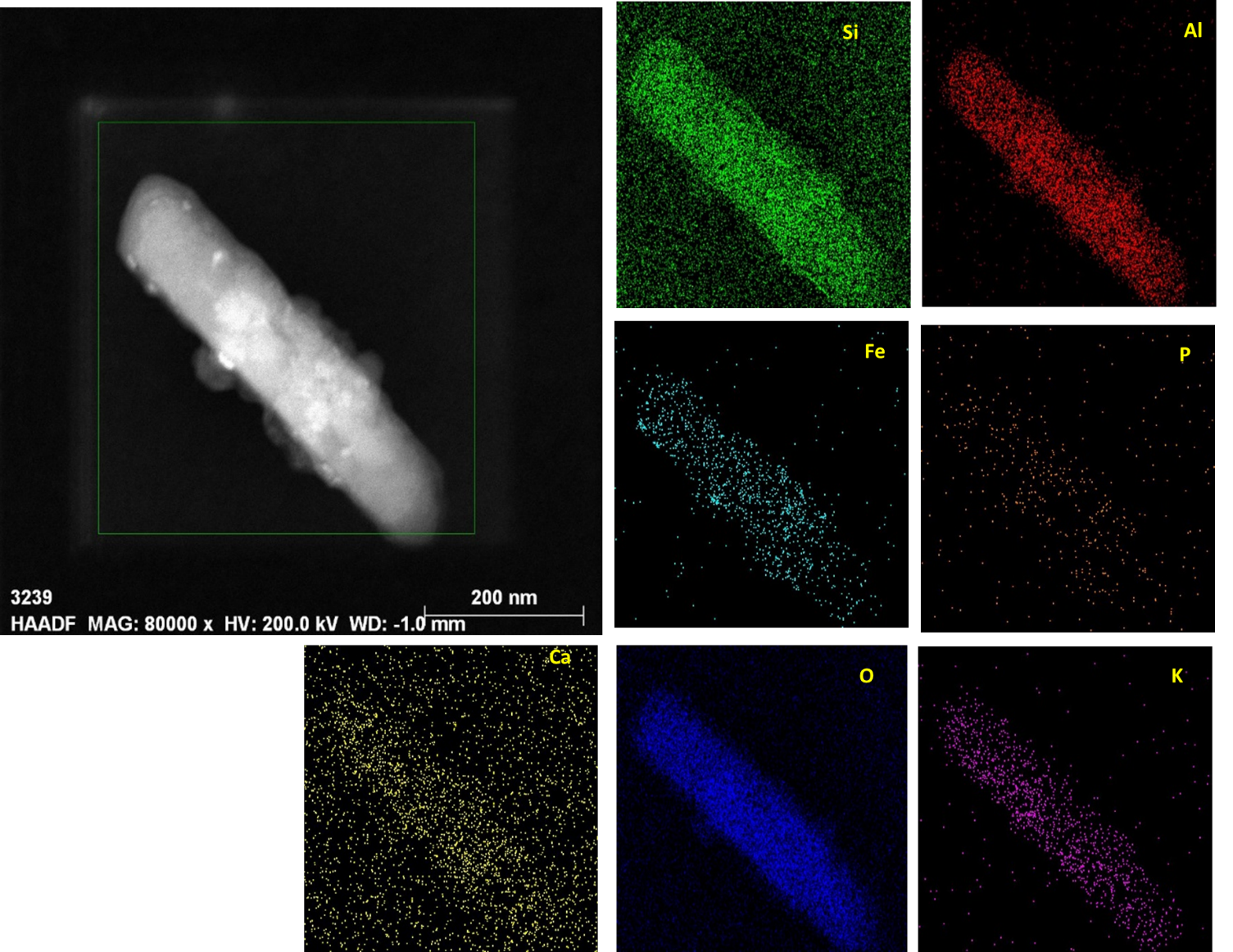


**Suppl. Figure 2**: STEM-EDS images of slag which is considered as a by-product of iron- making contains Si, Al, Fe, O, K, P, Ca.


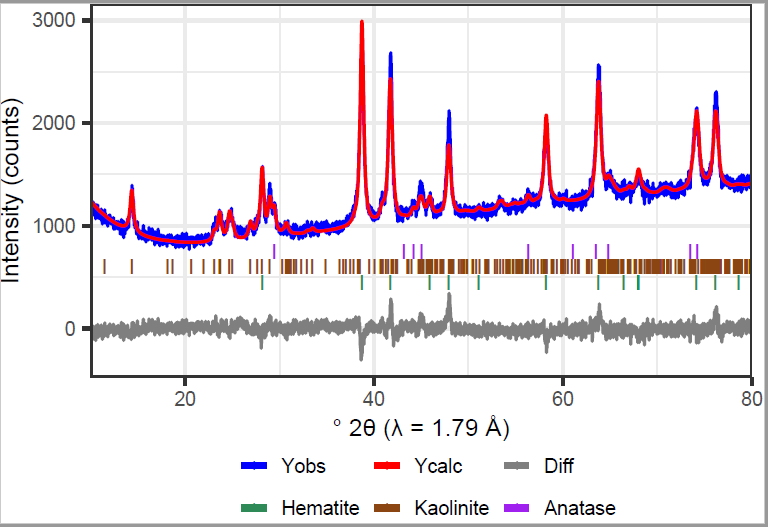


**Suppl. Figure 3**: XRD pattern of iron ore used in iron-making produced through ancient Indian metallurgical method depicting the presence of hematite, kaolinite and anatase phases.


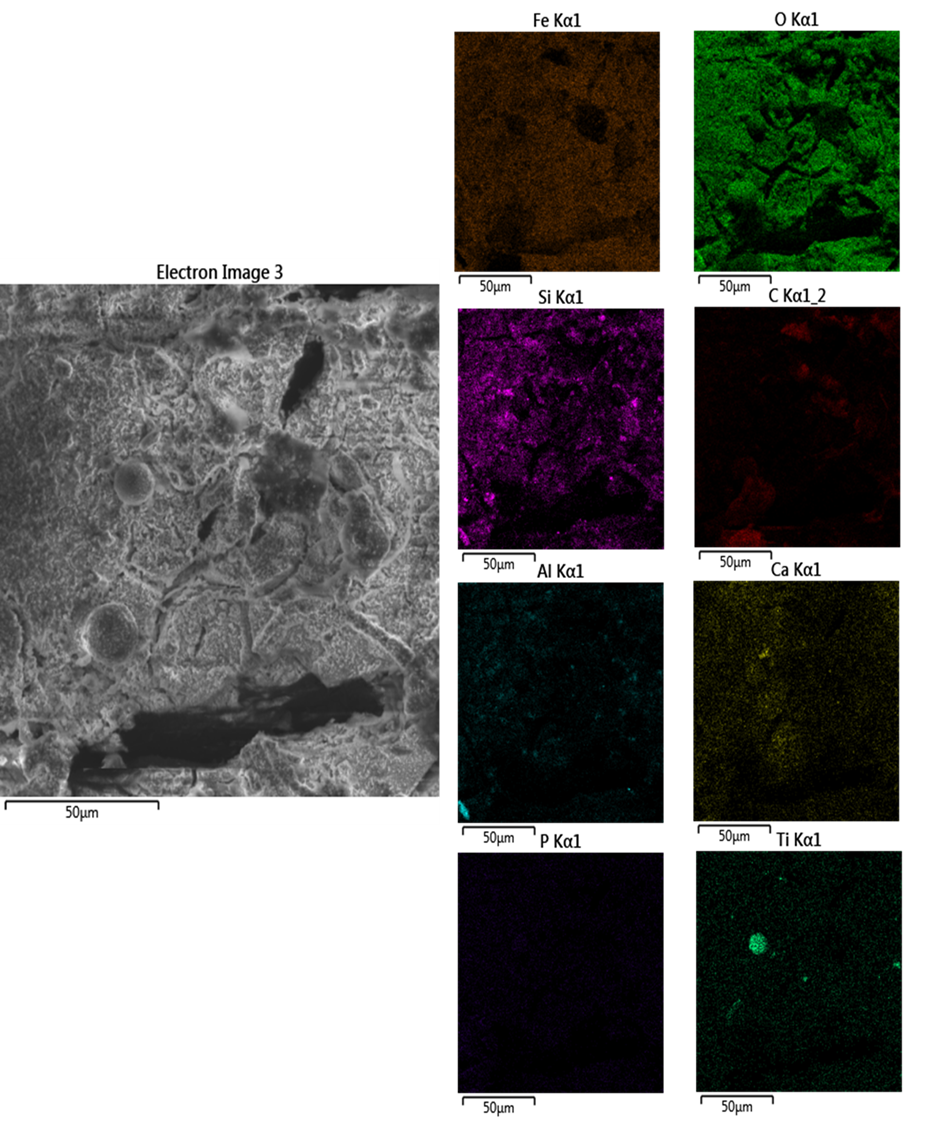


**Suppl. Figure 4**: FESEM-EDS mapping of corrosion product film formed on the surface of iron made through ancient Indian metallurgical method depicting presence of Fe, O, Si, C, Al, Ca, and Ti (Ti segregation is witnessed).


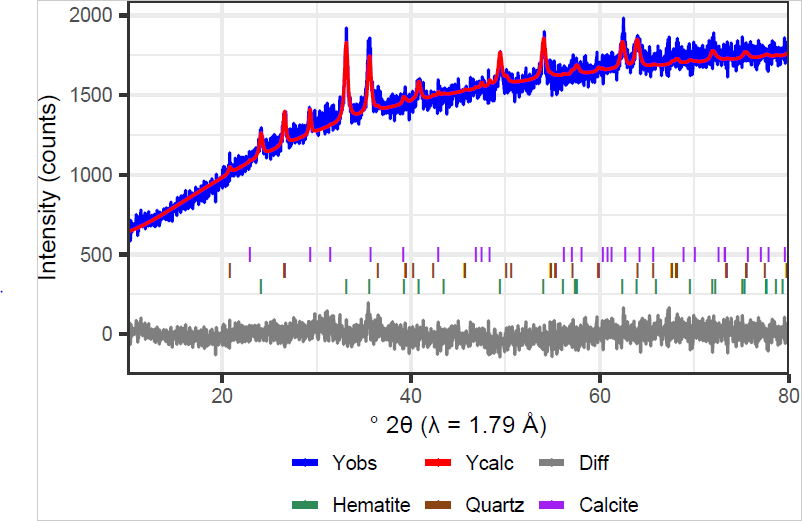


**Suppl. Figure 5**: GI-XRD pattern of corrosion product film formed on the surface of iron made through ancient Indian metallurgical method depicting the presence of hematite, calcite and quartz.


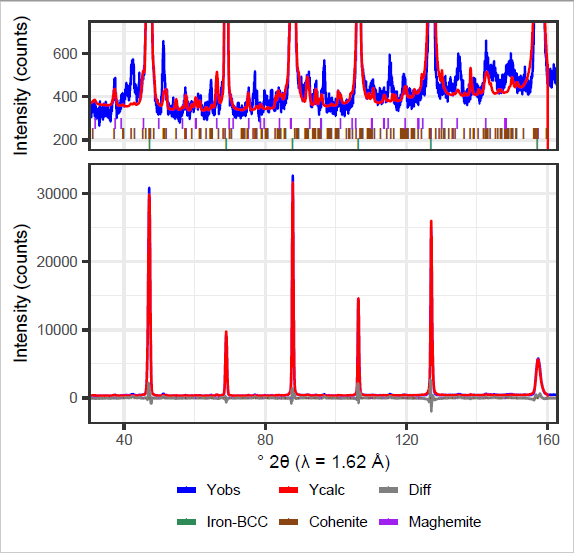


**Suppl. Figure 6**: Neutron diffraction pattern of iron made through ancient Indian metallurgical method.


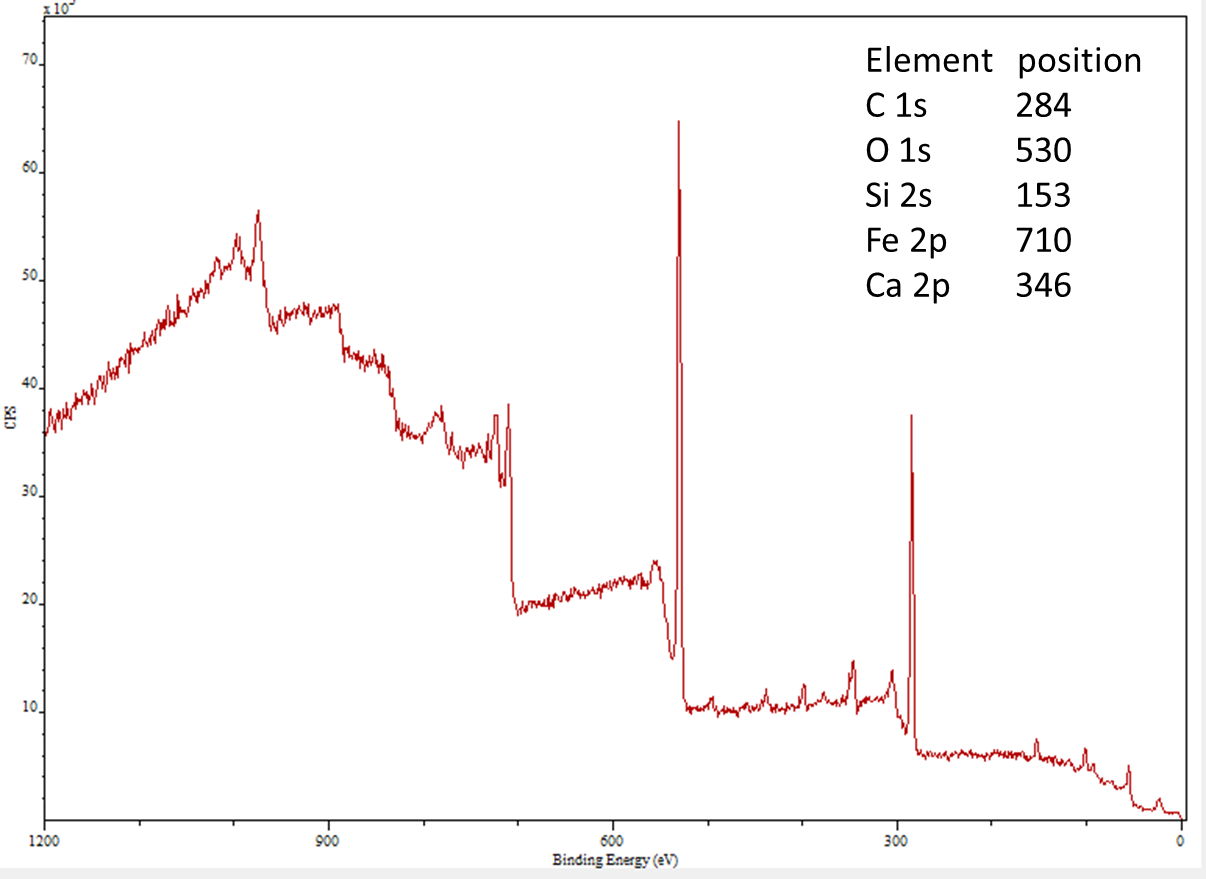


**Suppl. Figure 7**: X-ray photoelectron spectroscopic survey scan of corrosion product film formed on the surface of iron (un-etched) made through ancient Indian metallurgical method.


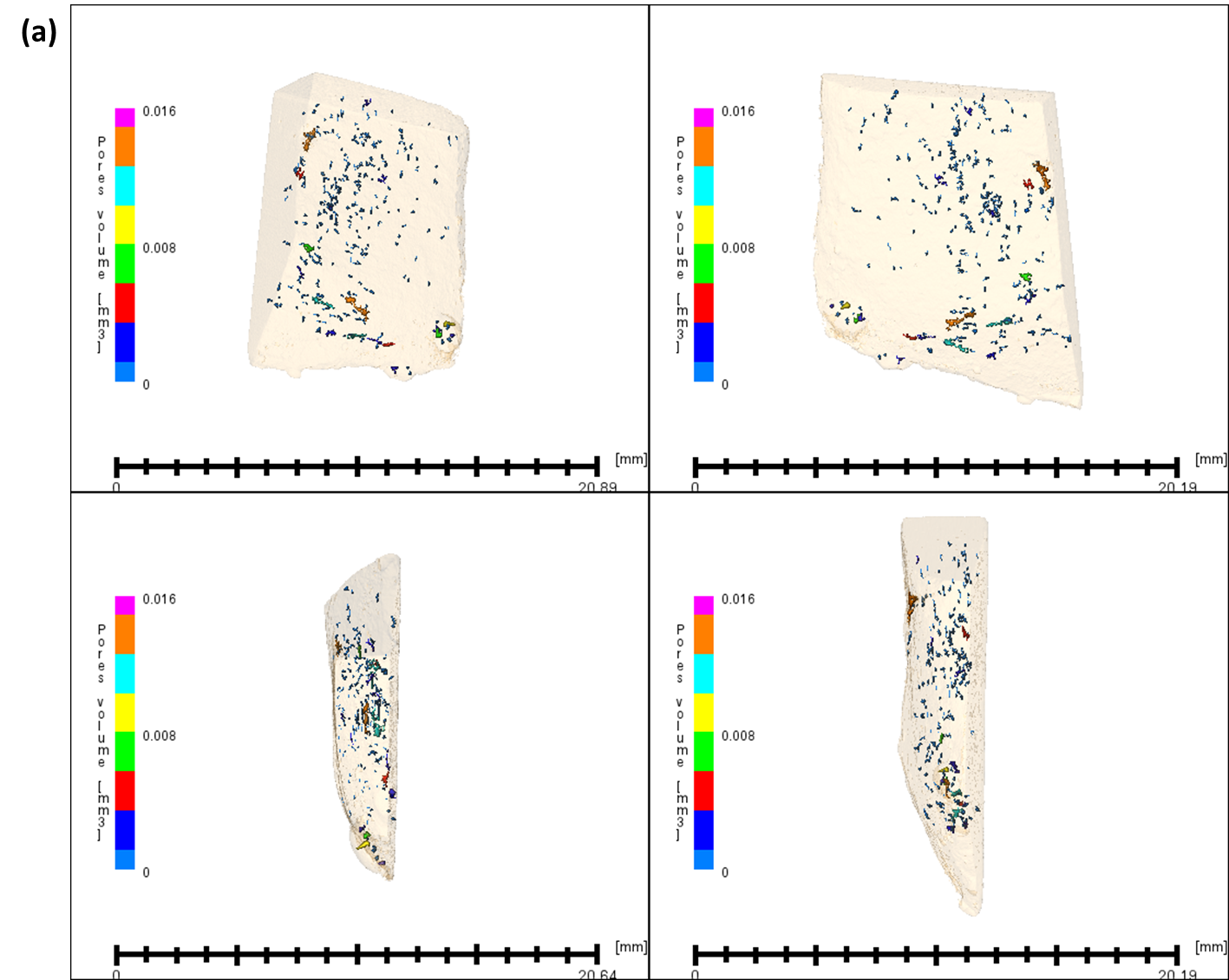


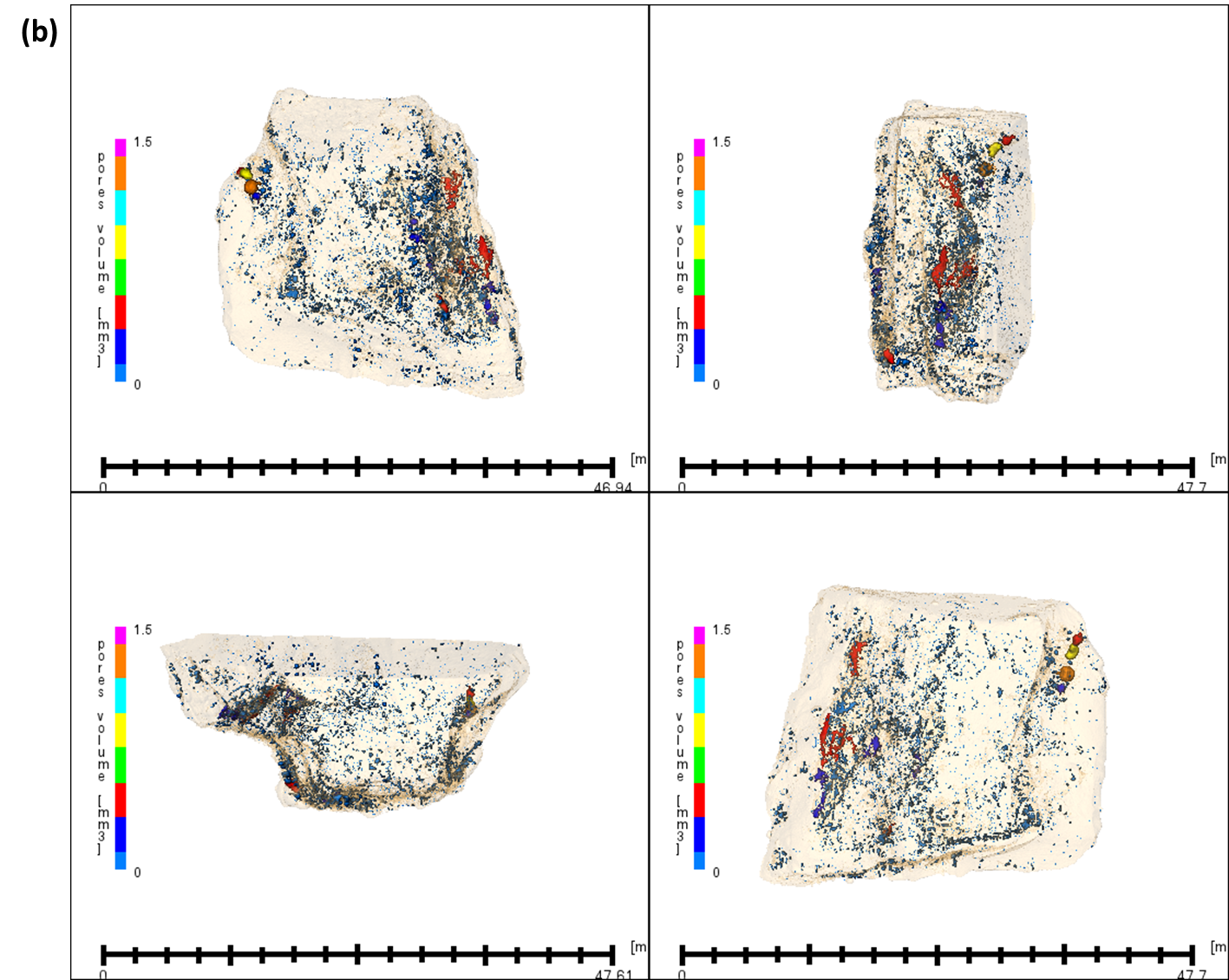


**Suppl. Figure 8**: Comparing the neutron tomographic images depicting the volume of the pores that are formed on the iron made through ancient Indian metallurgical method in (a) un-hammered iron (b) hammered (forged) iron from different orientations.


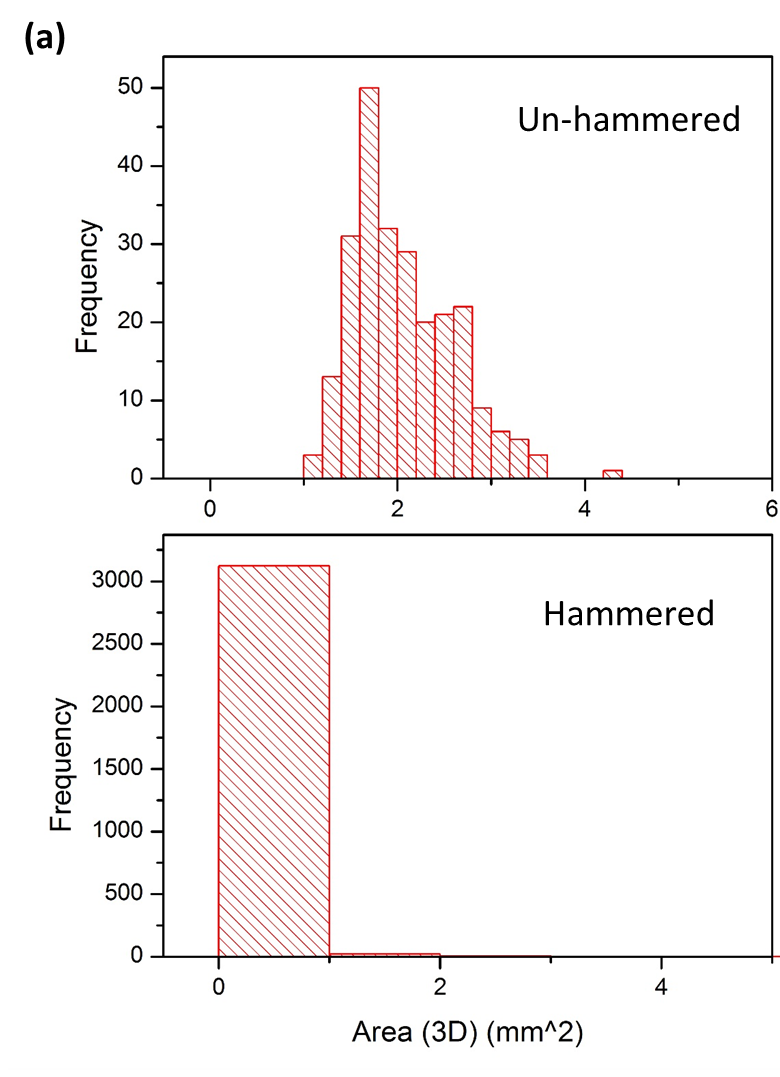

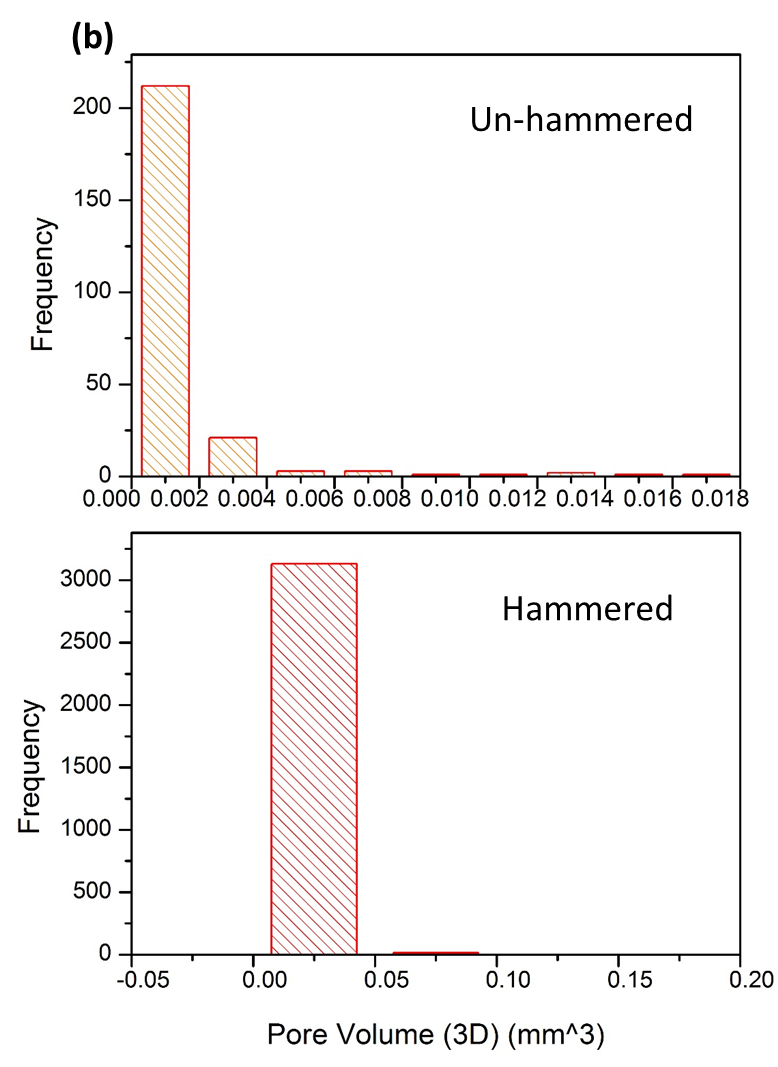


**Suppl. Figure 9**: Comparing the histograms of (a) area (in 3-D) and (b) volume (in 3-D) of pores present in the un-hammered and hammered iron made through ancient Indian metallurgical method of Agaria tribes. These results are obtained from the 3-D analysis of neutron tomographic images of un-hammered iron and hammered (forged) iron.


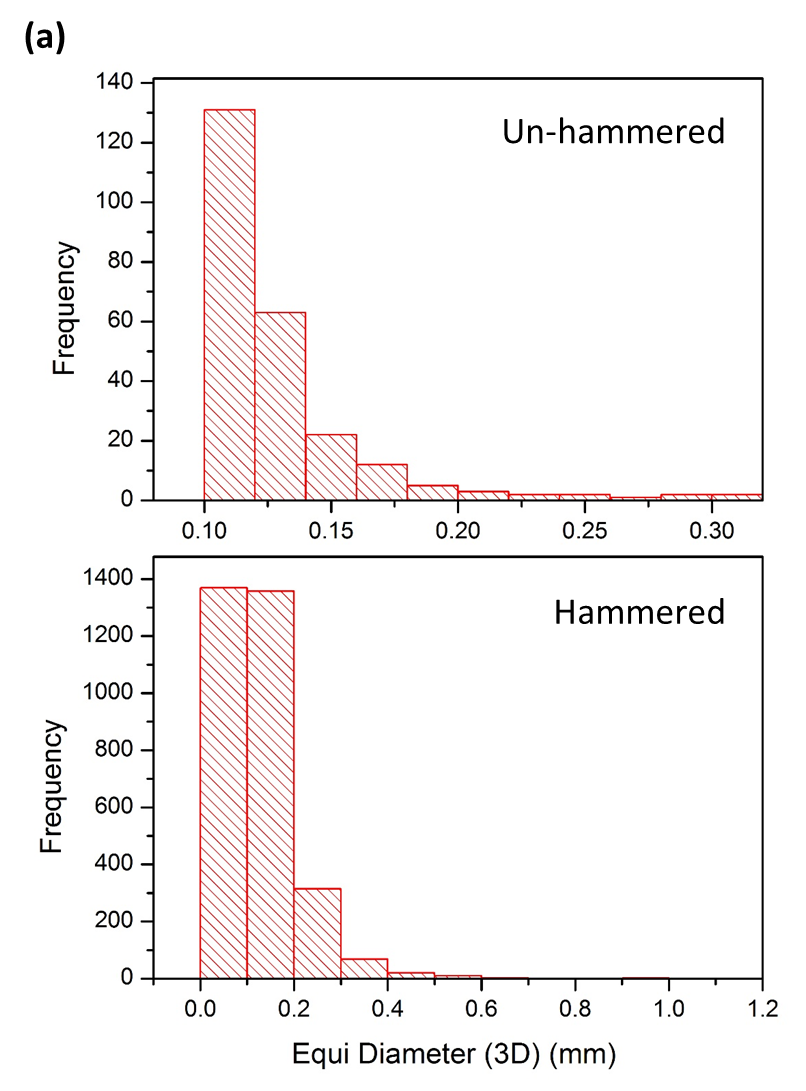


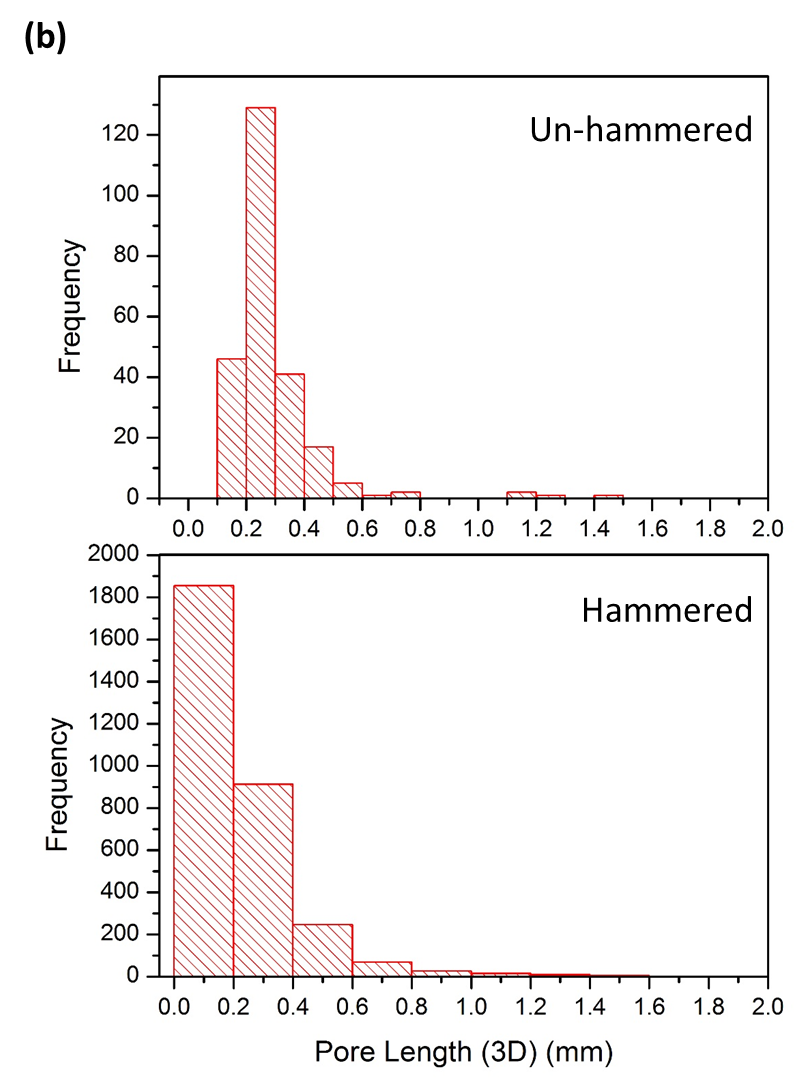


**Suppl. Figure 10**: Comparing the histograms of (a) equi-diameters (in 3-D) and (b) length (in 3-D) of pores present in the un-hammered and hammered iron made through ancient Indian metallurgical method of Agaria tribes. These results are obtained from the 3-D analysis of neutron tomographic images of un-hammered iron and hammered (forged) iron.


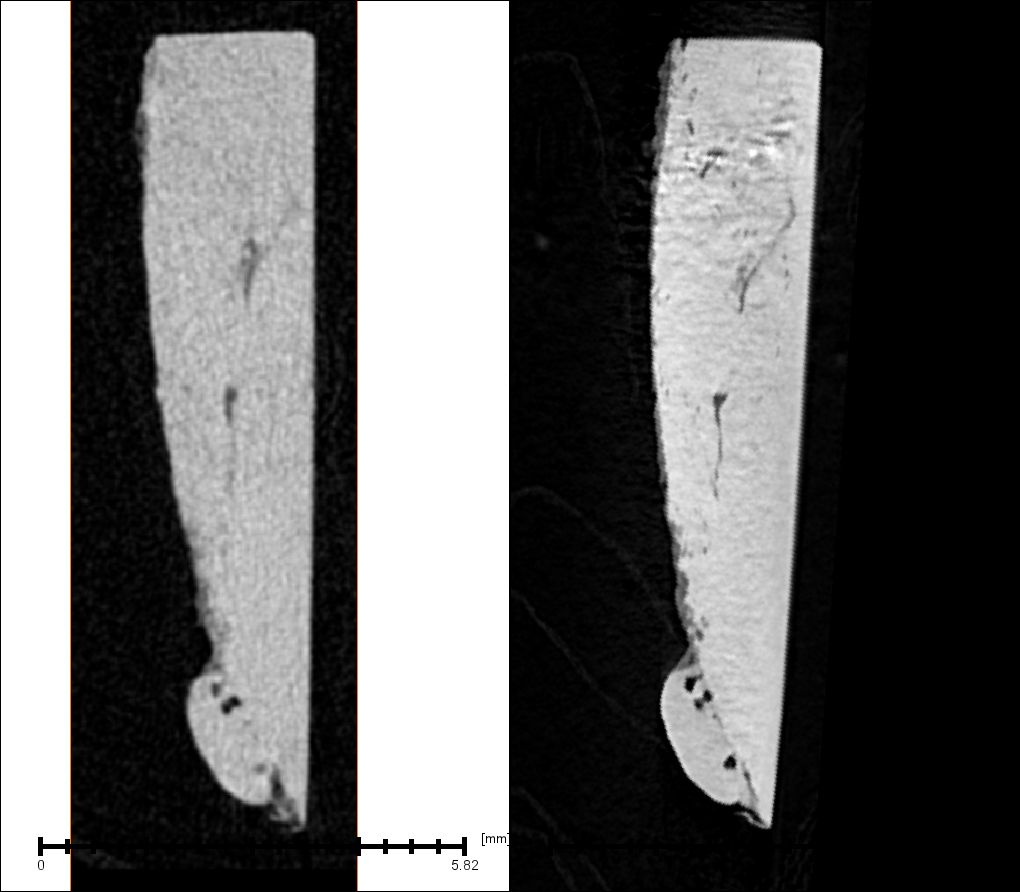


Artefact

**Suppl. Figure 11**: Neutron tomography (left) and synchrotron X-ray CT (right) image of iron made through ancient Indian metallurgical method depicting the instrumental artefacts development in synchrotron X-ray CT.


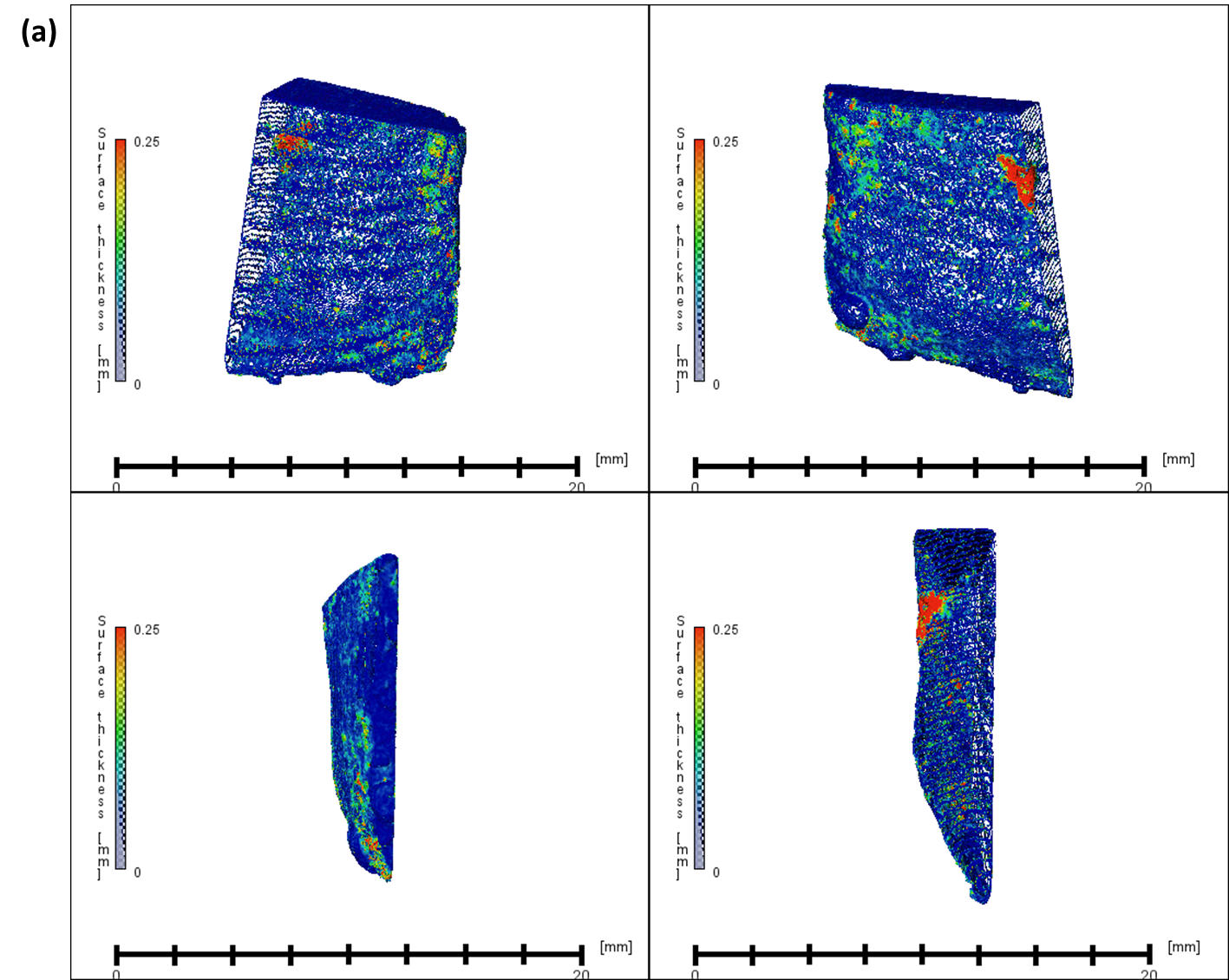


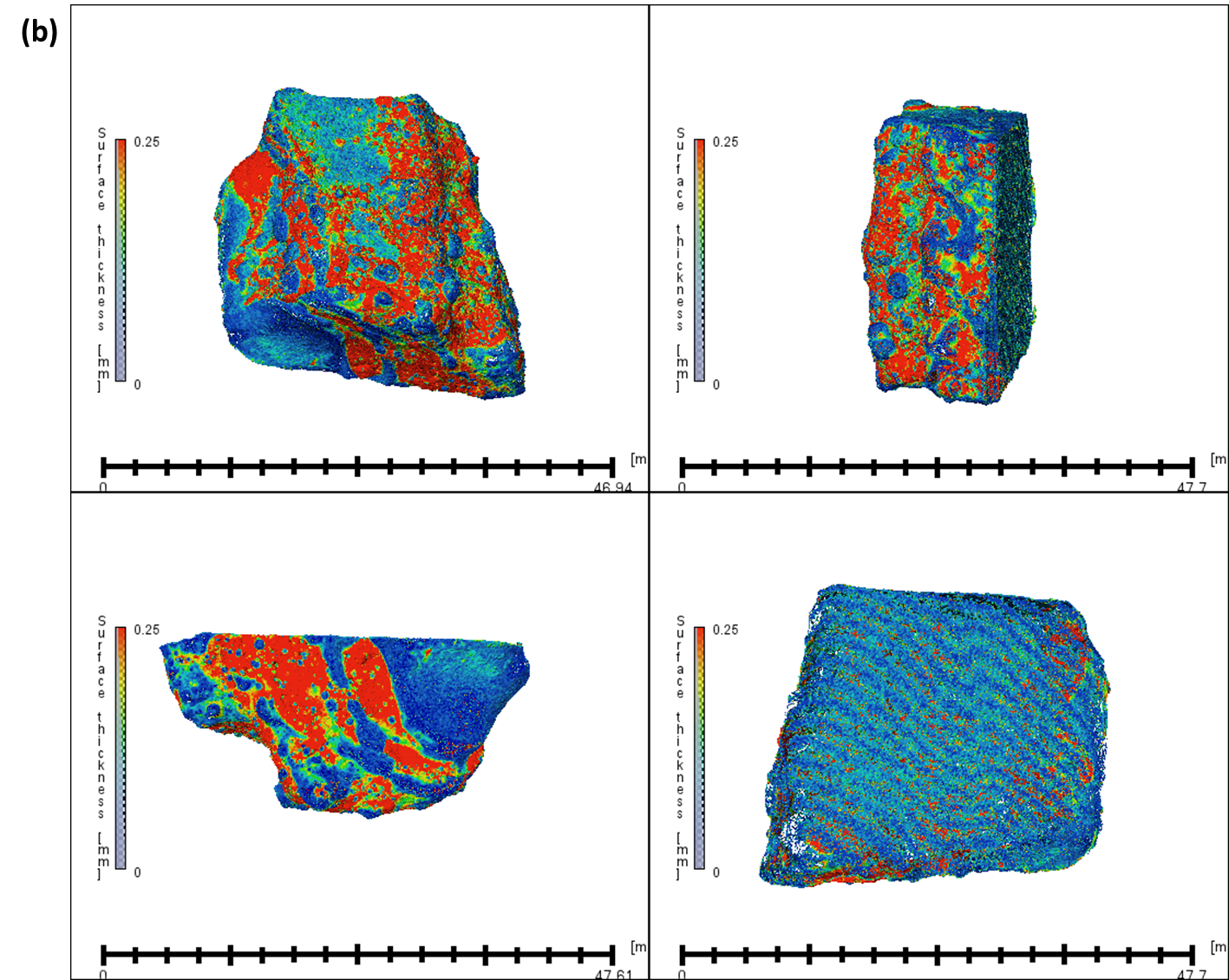


**Suppl. Figure 12**: Comparing the neutron tomographic images depicting the corrosion product film thickness that are formed on the iron made through ancient Indian metallurgical method in (a) un-hammered iron (b) hammered (forged) iron from different orientations.

***Theory of SANS*:**

Coherent scattering differential scattering cross-section per unit volume (*I(q)*) in SANS experiments can be expressed as a function of *q* and can be expressed as,

$I(q)=\emptyset V\left( \rho_{p} \right)^{2}P\left( q \right)S\left( q \right)+B$ (1)

In equation (1), volume fraction of particles and particle volumes are expressed by $\emptyset$ and $V$ respectively. On the other hand, scattering length densities which plays a major role on structure dependent models, is represented by$\rho_{p}$. Intraparticle and interparticle structure factors and represented by $P\left( q \right)$ (provides information about size and shape of the particle) and $S\left( q \right)$ respectively and incoherent background (of sample) is denoted by *B*.

Guinier-Porod model which is considered as shape independent model, is used for characterizing the film on iron made by Agarian tribes. For Guinier-Porod model, intensity can be defined by following equations:

$I\left( q \right)=G exp\left( \frac{q^{2}{R_{g}}^{2}}{3} \right)for q\leq q_{1,}$

$I\left( q \right)=\frac{D}{q^{m}} for q\geq q_{1}$ (2)

In equation (2), $R_{g}$ denotes radius of gyration and *m* depicts the Porod exponent. Similarly, *G* and *D* are the scale factors for Guinier and Porod respectively. Further details on this model is given elsewhere^1^.

**References:**

[1] Hammouda, B. A new Guinier–Porod model. *J. Appl. Crystallogr.* **43**, 716-719 (2010).
